# Dehydroabietylamine exerts antitumor effects by affecting nucleotide metabolism in gastric cancer

**DOI:** 10.1093/carcin/bgae037

**Published:** 2024-06-13

**Authors:** Jingsong Ma, Jiabao Zhao, Zhengxin Wu, Jinshui Tan, Meijuan Xu, Wenjie Ye, Mengya Zhong, Yubo Xiong, Guangchao Pan, Huiwen Zhou, Shengyi Zhou, Xuehui Hong

**Affiliations:** Department of Gastrointestinal Surgery, Zhongshan Hospital of Xiamen University, School of Medicine, Xiamen University, Xiamen 361000, China; Xiamen Municipal Key Laboratory of Gastrointestinal Oncology, Xiamen 361000, China; Department of Gastrointestinal Surgery, Zhongshan Hospital of Xiamen University, School of Medicine, Xiamen University, Xiamen 361000, China; Xiamen Municipal Key Laboratory of Gastrointestinal Oncology, Xiamen 361000, China; Department of Radiology, The First Affiliated Hospital of Zhejiang Chinese Medical University, Hangzhou 310006, China; Department of Gastrointestinal Surgery, Zhongshan Hospital of Xiamen University, School of Medicine, Xiamen University, Xiamen 361000, China; Xiamen Municipal Key Laboratory of Gastrointestinal Oncology, Xiamen 361000, China; Department of Gastrointestinal Surgery, Zhongshan Hospital of Xiamen University, School of Medicine, Xiamen University, Xiamen 361000, China; Xiamen Municipal Key Laboratory of Gastrointestinal Oncology, Xiamen 361000, China; Department of Gastrointestinal Surgery, Zhongshan Hospital of Xiamen University, School of Medicine, Xiamen University, Xiamen 361000, China; Xiamen Municipal Key Laboratory of Gastrointestinal Oncology, Xiamen 361000, China; Department of Hematology, The First Affiliated Hospital of Xiamen University and Institute of Hematology, School of Medicine, Xiamen University, Xiamen 361003, China; Department of Hematology, The First Affiliated Hospital of Xiamen University and Institute of Hematology, School of Medicine, Xiamen University, Xiamen 361003, China; Department of Gastrointestinal Surgery, Zhongshan Hospital of Xiamen University, School of Medicine, Xiamen University, Xiamen 361000, China; Xiamen Municipal Key Laboratory of Gastrointestinal Oncology, Xiamen 361000, China; Department of Gastrointestinal Surgery, Zhongshan Hospital of Xiamen University, School of Medicine, Xiamen University, Xiamen 361000, China; Xiamen Municipal Key Laboratory of Gastrointestinal Oncology, Xiamen 361000, China; Department of Gastrointestinal Surgery, Zhongshan Hospital of Xiamen University, School of Medicine, Xiamen University, Xiamen 361000, China; Xiamen Municipal Key Laboratory of Gastrointestinal Oncology, Xiamen 361000, China; Department of Gastrointestinal Surgery, Zhongshan Hospital of Xiamen University, School of Medicine, Xiamen University, Xiamen 361000, China; Xiamen Municipal Key Laboratory of Gastrointestinal Oncology, Xiamen 361000, China

## Abstract

Nucleotide metabolism is the ultimate and most critical link in the self-replication process of tumors, including gastric cancer (GC). However, in clinical treatment, classic antitumor drugs such as 5-fluorouracil (5-FU) are mostly metabolic analogs of purines or pyrimidines, which lack specificity for tumor cells and therefore have significant side effects. It is unclear whether there are other drugs that can target nucleotide metabolism, except for nucleic acid analogs. Here, we found that a natural compound, dehydroabietylamine (DHAA), significantly reduced the viability and proliferation of GC cells and organoids. DHAA disrupts the purine and pyrimidine metabolism of GC cells, causing DNA damage and further inducing apoptosis. DHAA treatment decreased transcription and protein levels of key enzymes involved in the nucleotide metabolism pathway, with significant reductions in the expression of pyrimidine metabolism key enzymes CAD, DHODH, and purine metabolism key enzymes PAICS. We also found that DHAA directly binds to and reduces the expression of Forkhead box K2 (FOXK2), a common transcription factor for these metabolic enzymes. Ultimately, DHAA was shown to delay tumorigenesis in *K19-Wnt1/C2mE* transgenic mice model and reduce levels of CAD, DHODH, and PAICS *in vivo*. We demonstrate that DHAA exerts an anticancer effect on GC by targeting transcription factor FOXK2, reducing transcription of key genes for nucleotide metabolism and impairing nucleotide biosynthesis, thus DHAA is a promising candidate for GC therapy.

## Teaser

Dehydroabietylamine affects protein levels of the transcription factor FOXK2 through direct interaction with FOXK2. It interferes with the transcription and expression of key genes in the nucleotide metabolism pathway, restricts nucleotide metabolism, and affects the growth of gastric cancer.

## Graphical Abstract

**Graphical Abstract:**
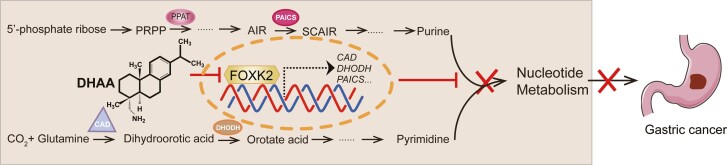

## Introduction

Gastric cancer (GC) is a malignant tumor with one of the highest mortality rates in the world [1, 2]. Although patients with early-stage GC have a good prognosis after radical surgery with chemotherapy, with a 5-year survival rate of up to 90%, many patients are already at an advanced stage at the time of initial diagnosis due to genetic heterogeneity and difficulties in early screening [3]. For metastatic and recurrent GC, chemotherapy has gradually become the accepted standard of care in clinical practice. 5-Fluorouracil (5-FU) and its derivatives (TS-1 and capecitabine) are the most widely used and studied antitumor drugs for GC and have long played a pivotal role in most chemotherapy regimens [4, 5]. However, acquired resistance to 5-FU is now becoming an increasing clinical problem.

5-FU is a pyrimidine analog that acts primarily as an inhibitor of thymidine synthase, blocking the synthesis of thymine, disrupting nucleotide metabolism, and ultimately inhibiting tumor progression [6]. All living cells need nucleotides as raw materials to synthesize DNA and RNA for transcription, translation, and various posttranslational modifications, which increase the biomass of the cell [7]. To avoid errors in these processes, nucleotide metabolic pathways are tightly regulated at various levels to maintain the stability of the adenine, guanine, cytosine, uracil, and thymine pools. Unrestricted proliferation of tumor cells is heavily dependent on nucleotide synthesis [8, 9]. The MYC family is a well-studied group of oncogenes, and members of this family are believed to regulate the proliferation, growth, and metabolism of many tumor cells by directly binding and increasing the expression of pivotal enzymes in nucleotide metabolism, including thymidylate synthase, inosine monophosphate dehydrogenase (IMPDH), and the trifunctional enzymes carbamoyl-phosphate synthetase 2, aspartate transcarbamylase, and dihydroorotase (CAD) [10–14]. Our previous research also found that U2AF homology motif kinase 1 can activate 5ʹ-aminoimidazole-4ʹ-carboxamide ribonucleic acid formyl transferase (ATIC) and IMPDH, the key enzymes in purine anabolism, through the NCOA3/ATF4 axis, promoting GC progression [15]. CDC-like kinase 3 can activate ATIC, the bottleneck enzyme in the *de novo* purine synthesis pathway, by regulating the USP13/Fbxl14/c-Myc signaling axis and promoting the occurrence of cholangiocarcinoma [16]. In hepatocellular carcinoma, dual-specific tyrosine phosphorylation-regulated kinase 3 downregulates the expression of 5ʹ-phosphoribose pyrophosphate aminotransferase (PPAT). PPAT is a rate-limiting enzyme in *de novo* purine synthesis that catalyzes 5ʹ-phosphoribo-1ʹ-pyrophosphate (PRPP) to 5ʹ-phosphoriboamine, thereby inhibiting cancer cell growth and metastasis [17]. From a disease perspective, unstrained proliferation is conducive to inducing mutations that promote the virulence and evolution of a disease, enabling it to survive in a changing environment [9]. Thus, targeting nucleotide metabolism to limit the uncontrolled replication of tumor cells is a promising strategy for treating cancer.

Dehydroabietylamine (DHAA), also called leelamine ((1,4*a*-dimethyl-7-propan-2-yl-2,3,4,9,10,10a-hexahydrophenanthren-1-yl)methanamine), a natural compound extracted from pine bark, has been reported to have antitumor effects on a variety of cancers in recent years [18]. Previous studies have shown that DHAA has a weak agonistic effect on cannabinoid receptors and inhibitory effects on pyruvate dehydrogenase kinases and acetylcholinesterase [18, 19]. Furthermore, DHAA has been shown to target key oncogenic pathways (RTK–AKT/STAT3/MAPK, AKT/mTOR) in various tumor cells [18]. In particular, it has attracted extensive attention due to its effects on lysosomes and its ability to effectively inhibit the proliferation and tumorigenesis of human melanoma, prostate cancer, and breast cancer cells [20–23]. To our knowledge, the effect of DHAA on GC is currently unknown and is worth studying.

Here, we explored the effect of DHAA on the viability of GC cells or organoids and discovered that DHAA could reduce the viability and inhibit the proliferation of GC cells or organoids. Next, through RNA sequencing, we found that DHAA could affect purine and pyrimidine metabolism in GC cells simultaneously. Mechanically, DHAA can directly bind to and reduce the expression of FOXK2, a common transcription factor for multiple nucleotide-metabolizing enzymes. This result was verified by Western blotting and quantitative real-time (qRT)-PCR. *In vivo* experiments also demonstrated that DHAA-treated *K19-Wnt1/C2mE* transgenic mice had fewer malignant tumors and lower expression levels of several key enzymes in nucleotide metabolism than untreated *K19-Wnt1/C2mE* transgenic mice. In conclusion, our research indicates that DHAA inhibits tumor proliferation by affecting nucleotide metabolism, thereby delaying GC progression.

## Materials and methods

### Cell lines, culture conditions, and reagents

Human GC cell line HGC-27 was purchased from the American Type Culture Collection (ATCC, Manassas, VA, USA) in July 2020. MGC-803 was kindly provided by Xiamen Municipal Key Laboratory of Gastrointestinal Oncology (Xiamen, China) in November 2020. Each cell line was identified by polymorphic short tandem repeat profiling in advance and tested every month to exclude mycoplasma contamination. October 2023 was the last time the cell lines were tested.

Both cell lines were cultured in Dulbecco’s modified Eagle’s medium (DMEM, Gibco, CA, USA) supplemented with 10% fetal bovine serum (Gibco, CA, USA) and 100 U/ml penicillin and streptomycin (Gibco, CA, USA) in a humidified environment containing 5% CO_2_ at 37°C.

DHAA (Cat# BD123672) was purchased from Bidepharm (Shanghai, China) and dissolved in sterile DMSO (Sigma, MO, USA) to prepare a 50 mM stock solution, which was divided into 200 µl aliquots and stored at −20°C for later use.

### Organoid culture

We constructed and cultured organoids based on a report by Bartfeld *et al*. [24, 25]. Briefly, we cut approximately 1 cm of tumor tissue into small pieces, digested the tissue, washed it with Advanced DMEM F12 medium (Gibco, CA, USA), seeded it on Matrigel (Corning, New York, USA), and treated it with penicillin, streptomycin, GC organoid medium (Five Elements Bio-Technology, Nanchang, China) and other factors. The organoids were cultured in a humidified environment containing 5% CO_2_ at 37°C.

### Measurement of cell and organoid viability

Cell viability was determined by the CCK-8 assay (APExBIO, Texas, USA) according to the manufacturer’s instructions. In short, 5 × 10^3^ cells were resuspended in 100 µl medium and then seeded in 96-well plates. When the cells were confluent, drugs were added. DMSO was used as the control. After culturing for 24 h or 48 h according to the experimental requirements, 10 µl CCK-8 reagent was added, the cells were cultured for approximately 1–2 h, and the absorbance was measured at 450 nm. We added 4 µM DHAA to the cultured organoids and observed the morphology of the organoids after 0 h, 24 h, 48 h, and 72 h under a microscope (Leica, Wetzlar, Germany). Organoid viability was assessed using CellTiter-Glo® 3D at the end of the incubation period (Promega, Madison, Wisconsin, USA).

### Colony formation assay

The colony formation assay was carried out on monolayer cultures. A total of 1 × 10^3^ cells per well were seeded in six-well cell culture plates for 2 weeks or until colonies were visible to the naked eye. The medium was replaced with fresh medium every 3 days on average. After fixation and staining, colonies were imaged and counted. Each experiment was repeated three times.

### RNA sequencing

HGC-27 cells were treated with DMSO and 4 µM DHAA for 24 h, and then total RNA was extracted with TRIzol reagent (TransGen Biotech, Beijing, China). In accordance with the manufacturer’s instructions, next-generation sequencing was performed to establish a library with an Illumina HiSeq-2500 instrument (Illumina, CA, USA) with different indexes. KEGG pathway analysis and GO enrichment analysis were carried out via R Studio software. The DESeq2 Bioconductor package was utilized to identify differentially expressed genes. The relationships between pathways were analyzed by employing the ClueGo plug-in in Cytoscape software. An adjusted *P* value <.05 was considered to indicate statistical significance.

### RNA extraction and qRT-PCR

Based on the manufacturer’s protocols, RNA was extracted from GC cells and tissues utilizing a TRIzol reagent. The cDNA Synthesis Supermix kit was used to reverse transcribe the RNA into cDNA. 2 × TransStart® Top/Tip Green qPCR SuperMix was employed to perform real-time PCR on a Bio-Rad Biosystems 7500 instrument (Bio-Rad, Hercules, CA). Three replicate wells were set up for each sample, and the experiment was repeated three times. All reagents used for qRT-PCR were purchased from TransGen Biotech in Beijing, China. Target RNA levels were normalized to β-actin levels. The primer sequences were as follows: CAD-F: GAAGAATATCCTGCTGACCATTGG, CAD-R: GAGACTGGCATAGAGGCTGTAG;

DHODH-F: CAATGCCTTACTCTGTGATTCG, DHODH-R: CTAATGACTTTGGGGTTACTGC;

UMPS-F: CATTGTAGGTCGTGGCATAATCTC, UMPS-R: CCAAGTCTACTCAAATACGCTTCC; PPAT-F: AAGTGCTGCTCTAAGTGGTTAT, PPAT-R: GCACTATCTCCAGAAATACCGA; GART-F: CTCCAGGTCAATCATCTTCTGT, GART-R: GAATCATGCCAGTGCTGTATTT; PFAS-F: AAGAAAAAGTGGGTAATGGTGC, PFAS-R: GCTCACCTATCTCTCTGAACTC; PAICS-F: ACAATGATTCCTGGAGACTCTGG, PAICS-R: TTTCTTTACCATTTGGAGCCCTTC; ADSL-F: TTGGTGCTACTTCTTGCTATGTTG, ADSL-R: GGTAGACTGGCTCGTTCCTTAG; ATIC-F: GAACCTCGAGCTGATCTTCG, ATIC-R: CTGAGCTCCGATTCGAAGG; ADSS1-F: GTGACCACAGGCAGGAAGAG, ADSS1-R: GCAGTGAATCCGTTGACCATG; ADSS2-F: CTTCCTGACCCTATGTCCAGAG, ADSS2-R: CTGCATGAGTCTCTCCTTGTC; APRT-F: GGTGGTCGTCGTGGATGATC, APRT-R: GGTACAGGTGCCAGCTTCTC; IMPDH1-F: GAAGACGCCACTGATCTCCTC, IMPDH1-R: TTCTTGACCTTCCGCACCTC; IMPDH2-F: GGACCTGACTTCTGCTCTGAC, IMPDH2-R: AGCCAATACCGCCTGTAAGC; GMPS-F: AGTTGGGCTTAGAGGCTATTTT, GMPS-R: ATTGTGGTCTGAAAACCGATTG; β-actin-F: GGACTTCGAGCAAGAGATG, β-actin-R: AGCACTGTGTTGGCGTACAG.

### Western blot analysis

GC cells were placed in 10 cm culture dishes, and when the cells adhered to the walls of the dish and stretched, drugs were added. DMSO was used as the group. After 24 h of culture, an appropriate amount of lysis buffer containing protease and phosphatase inhibitors was added, and the cells were collected by scraping and placed in a vertical rotator at 4°C for 30 min. The supernatant was collected by centrifugation at 4°C. The protein was then quantified by the BCA assay (Thermo Scientific, MA, USA). An sodium dodecyl sulfate polyacrylamide gel electrophoresis (SDS‒PAGE) gel was prepared, and the proteins were separated by electrophoresis (Bio-Rad, CA, USA). The proteins were transferred to a polyvinylidene fluoride (PVDF) membrane. After blocking, the membrane was incubated with primary antibody overnight at 4°C and then with secondary antibody at room temperature for 1–2 h. Finally, ClarityTM Western ECL Substrate (Bio-Rad, CA, USA) was used to visualize the bands. Anti-CAD (Cat# 11933, 1:1000), anti-IMPDH2 (Cat# 36281, 1:1000), anti-GMPS (Cat# 14602, 1:1000), anti-DYKDDDDK (Cat# 8146, 1:1000), anti-c-Myc (Cat# 5605, 1:1000), anti-p65 (Cat# 8242, 1:1000), anti-MITF (Cat# 12590, 1:1000), anti-ATF4 (Cat# 11815, 1:1000), and anti-β-actin (Cat# 3700, 1:1000) antibodies were purchased from Cell Signaling Technology. Anti-DHODH (Cat# A13295, 1:1000), anti-UMPS (Cat# A13251, 1:1000), anti-PPAT (Cat# A6698, 1:1000), anti-GART (Cat# A3876, 1:1000), anti-PFAS (Cat# A17517, 1:1000), anti-PAICS (Cat# A6450, 1:1000), anti-ADSL (Cat# A6278, 1:1000), anti-ATIC (Cat# A5559, 1:1000), anti-ADSS1 (Cat# A0178, 1:1000), anti-ADSS2 (Cat# A12398, 1:1000), anti-APRT (Cat# A5456, 1:1000), anti-SP1 (Cat# A14662, 1:1000), anti-E2F1 (Cat# A16720, 1:1000), anti-FOXK2 (Cat# A14245, 1:1000), and anti-IMPDH1 (Cat# A16899, 1:1000) antibodies were purchased from ABclonal. Anti-rabbit secondary antibody (Cat# ab150077, 1:1000) was purchased from Abcam, and an anti-mouse secondary antibody (Cat# 1706516, 1:1000) was purchased from Bio-Rad.

### Apoptosis assay

The detected cells were treated with DHAA or DMSO and then harvested using Trypsin-EDTA. After washing with 1 × PBS twice, the cells were resuspended in 1 × binding buffer and incubated with PI and Annexin VFITC obtained from Annexin V FITC/PI Apoptosis Detection Kit (Yeasen). After staining the cells at room temperature for 15 min in the dark, cells were immediately stored in ice and analyzed using flow cytometry (Cyto-FLEXS) within 1 h, according to the manufacturer’s instructions.

### EdU incorporation assay

According to the manufacturer’s instructions, cell cycle analysis was carried out using the BeyoClickTM EdU Cell Proliferation Kit with Alexa Fluor 647 (Beyotime, Shanghai, China) and propidium iodide (Pl)/RNase Staining Buffer. In summary, 10 μM 5-ethyny1-2ʹ-deoxyuridine was incubated with drug-treated cells for 1 hour. Following incubation, the cells were collected, cleaned with PBS, and fixed in 70% ethanol for 12 h at 4°C. Data were analyzed using FlowJo software after the addition of the PI reagent and flow cytometry. The results are presented as the mean ± SD, and each experiment was run at least three times.

### Plasmid construction

The cDNA encoding human SP1 (FN908228), E2F1 (M96577), and FOXK2 (X60787) were subcloned into EcoR I/Xho I sites of the pCMV-C-3xFlag expression vector (Public Protein/Plasmid Library).

### *In vivo* experiments

K19-Wnt1/C2mE transgenic mice were obtained from Professor Masanobu Oshima’s laboratory. All animal experiments were performed based on the guidelines of the Animal Care and Use Committee and Ethics Committee of Xiamen University. As previously reported [26], 28-week-old mice (30–35 g) were used for *in vivo* experiments. The mice were randomly split into two groups (5 mice per group) and intraperitoneally injected with 200 μL vehicle (20% dimethyl sulfoxide, 30% Cremophor EL, and 50% phosphate-buffered saline) or DHAA (0.244 mg/mouse, equates to approximately 7.5 mg/kg body weight assuming an average body weight of 32.5 g) two times/week (Monday and Thursday) for four successive weeks. After 15 days of drug administration, the mice were euthanized. Micro-CT was performed first, and the presence of a tumor was confirmed by a Siemens Inveon PET/CT Multimodality System for laboratory animal CT. Then, gastric tissue was removed, and the tumor size was calculated by taking pictures. Tumor volumes were calculated on the basis of the formula *V* = (*L* × *W*2)/2 [*V*, volume; *L*, length; *W*, width]. Later, normal and tumor tissues were collected, fixed with 4% paraformaldehyde, stained with hematoxylin–eosin (H&E), and subjected to IHC staining.

### Histological analysis

Normal and tumor tissues were fixed, embedded in paraffin, and sectioned (4 μm). A portion was used for H&E staining. After dewaxing and rehydration, the other part was subjected to antigen retrieval (Maxim, DIG-3009, Fujian, China). Subsequently, reagents from an UltraSensitive S-P kit (Maxim, KIT-9730, Fujian, China) were utilized to block endogenous peroxidase activity. The tissues were incubated with the following primary antibodies overnight at 4°C: anti-CAD (Cat# 11933, 1:800, CST), anti-DHODH (Cat# A13295, 1:200, ABclonal), anti-PAICS (Cat# A6450, 1:200, ABclonal), anti-cleaved PARP (Cat# 5625, 1:100, CST), anti-CK8 (Cat# ab53280, 1:400, Abcam), anti-PCNA (Cat# ab18197, 1:1000, Abcam), anti-Ki67 (Cat# 48871, 1:400, Signalway Antibody), and anti-p53 (Cat# F2117, 1:500, Santa Cruz Biotechnology). They were then incubated with corresponding secondary antibodies the next day. An enhanced DAB chromogenic kit (Maxim, DAB-2032, Fujian, China) was utilized for detection. Finally, the sections were stained with hematoxylin and hydrochloric acid/ethanol, mounted with neutral resin, and observed under a microscope.

### Molecular docking

The FOXK2 protein structure was obtained from the AlphaFold Protein Structure Database (https://alphafold.ebi.ac.uk/) [27]. Maestro 11.1 software was employed to dock. Protein was considered rigid and small molecules were flexible during the docking process. Molecular docking results are presented in the 2D format using LigPlot.

### Statistical analysis

Unpaired Student’s *t*-test was utilized to examine the significance of the differences between groups. GraphPad Prism 8.0.1 software was employed for statistical analysis. A *P* value <.05 was considered to indicate statistical significance.

## Results

### DHAA reduces the viability and inhibits the proliferation of GC cells and organoids

**Figure 1:**
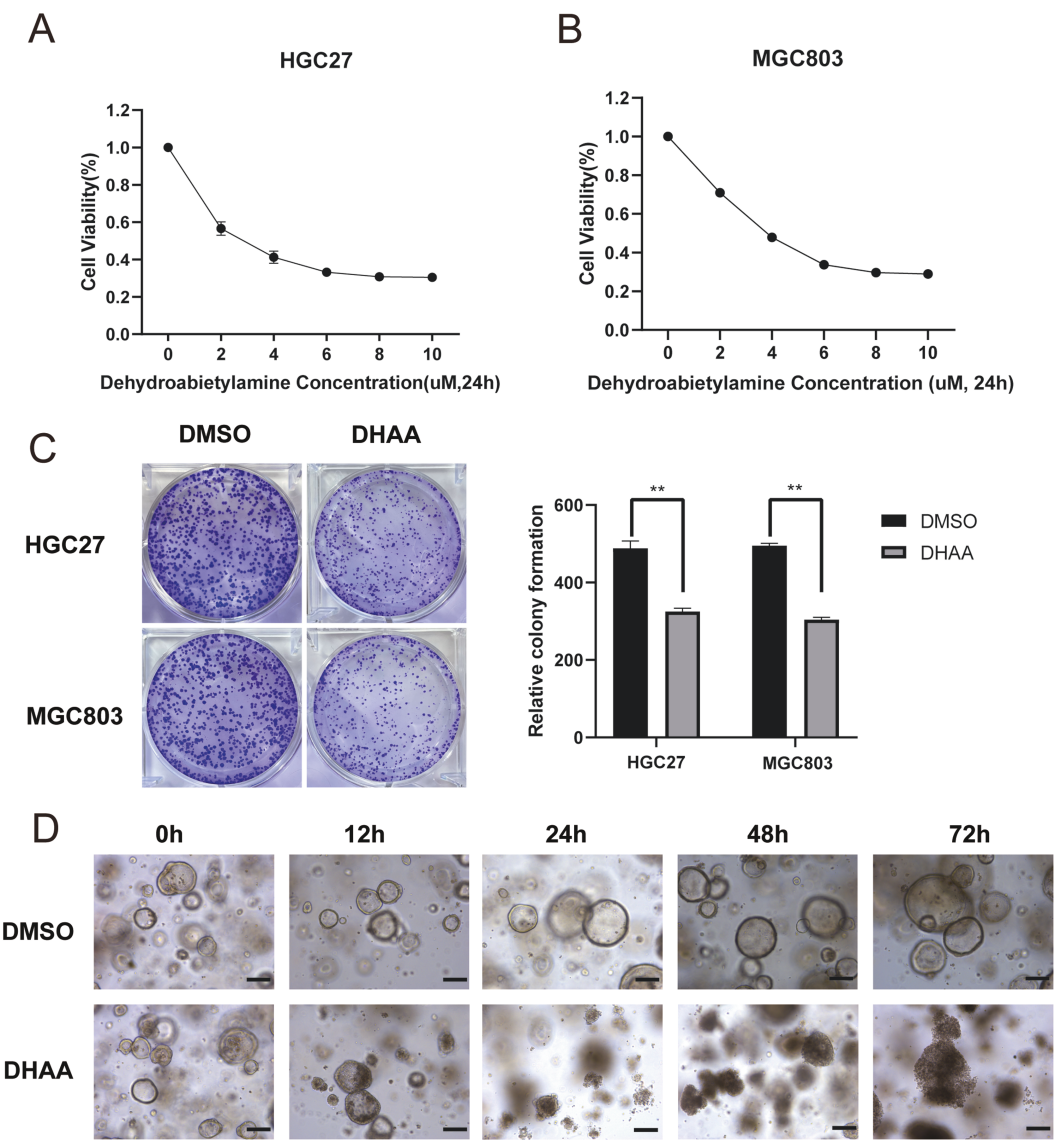
DHAA decreases the viability and proliferation of GC cells and organoids. (A, B) The viability of HGC-27 and MGC-803 cells was estimated utilizing the CCK-8 assay. (C) Quantitative analysis of the colony numbers of monolayer cells (images recorded under macro view). (D) Morphological observation of organoids under an electron microscope (**P < .01, scale bars, 25 μm).

To investigate the cytotoxic and inhibitory effects of DHAA (Supplementary Fig. 1A), the GC cell lines HGC-27 and MGC-803 were used in our study. The cell counting kit-8 (CCK-8) assay showed that DHAA (0-10 μM, 24 h) dose-dependently decreased GC cell viability, with 50% inhibitory concentrations (IC_50_) of 3.10 μM and 4.22 μM in HGC-27 and MGC-803 cells, respectively (Fig. 1A and B). Next, to test whether DHAA influences the proliferation of GC cells, a colony formation assay was conducted. After treatment with 4 µM DHAA, the efficiency of colony formation was markedly reduced, indicating that DHAA inhibited the proliferation of GC cells (Fig. 1C).

Compared to conventional 2D cultured cells, 3D cultured organoids exhibit a more complex physical link, promoting closer exchange of nutrients, and biological communication [28]. Induction, interaction, feedback, and cooperation between cells lead to the development and formation of functional micro organs or tissues [29]. 3D organoids can be utilized to more accurately simulate the physiological and pathological processes that occur in organs and tissues. We therefore used organoids for our experiments. The cell viability was markedly decreased in a dose-dependent manner assessed by the Cell Titer-Glo 3D reagent, indicating a significant inhibitory effect of DHAA on the growth of GC organoids, and the half-maximal inhibitory concentration (IC_50_) of DHAA was approximately 3.918 µM, which is comparable to the concentration in cell lines (Supplementary Fig. 1B). We added 4 µM DHAA to cultured organoids and observed the morphology of the organoids after 0 h, 24 h, 48 h, and 72 h. At 24 h, the proliferation of organoids was significantly inhibited in the DHAA group compared with the control group, which was treated with DMSO. After 48 h and 72 h, DHAA-treated organoids were completely disrupted and lysed (Fig. 1D). In conclusion, DHAA treatment can significantly reduce the viability and inhibit the proliferation of GC cell lines and organoids.

### Nucleotide metabolism contributes to the effect of DHAA in reducing the viability and inhibiting the proliferation of GC cells

**Figure 2:**
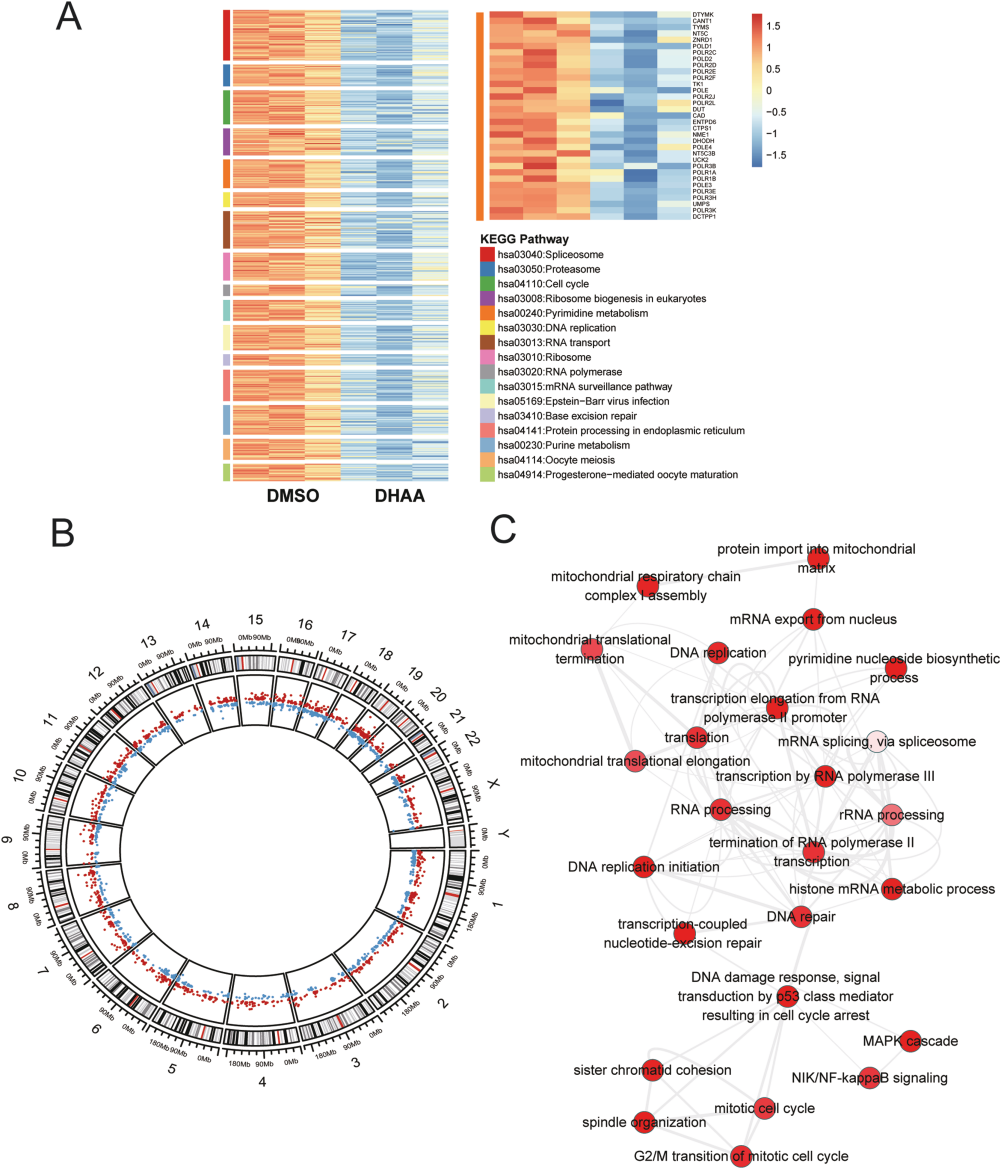
Effects of DHAA on genome-wide gene expression and related pathways in GC cells. (A) KEGG pathway analysis revealed that the differentially expressed genes were enriched in the nucleotide metabolic pathway and DNA replication. (B) Circle diagram of the differentially expressed genes after 4 μM DHAA treatment. (C) Analysis of the relationships between pathways associated with the differentially expressed genes.

To further explore the intrinsic mechanism by which DHAA reduces the viability and inhibits the proliferation of GC cells, RNA sequencing was performed. By analyzing the sequencing results and performing KEGG pathway enrichment analysis, we found that purine metabolism and pyrimidine metabolism were simultaneously decreased in GC cells after DHAA treatment, and the activity of signaling pathways such as DNA replication and the cell cycle was decreased (Fig. 2A). As shown in the right panel of Fig. 2A, the expression of genes in the purine metabolic and pyrimidine metabolic pathways was downregulated in cells treated with DHAA compared with control cells. The distribution of the differentially expressed genes on each chromosome is shown (Fig. 2B). The functions of the identified differentially expressed genes were explored, and Cytoscape software was employed to find the biological process interactions among the differentially expressed genes (Fig. 2C). The results also showed that pathways related to nucleotide metabolism, such as DNA replication, DNA repair, pyrimidine nucleoside biosynthesis, and RNA polymerase transcription, were significantly enriched. We wanted to investigate whether DHAA had the same effect on organoid biological function, and by analyzing organoid RNA sequencing results, we found that these findings also occurred in organoids, including DNA damage and nucleotide metabolic pathways from GO functional enrichment analysis (Supplementary Fig. 1C–F). Analysis of the KEGG pathway and biological interactions between the differentially expressed genes identified indicated that DHAA functions consistently in both cell lines and organoids (Supplementary Fig. 1G–I). Therefore, we speculate that DHAA may affect functions such as DNA replication by interfering with nucleotide metabolism in GC cells and organoids, thereby reducing the viability and inhibiting the proliferation of GC.

### DHAA induces DNA damage and apoptosis in GC cells

**Figure 3:**
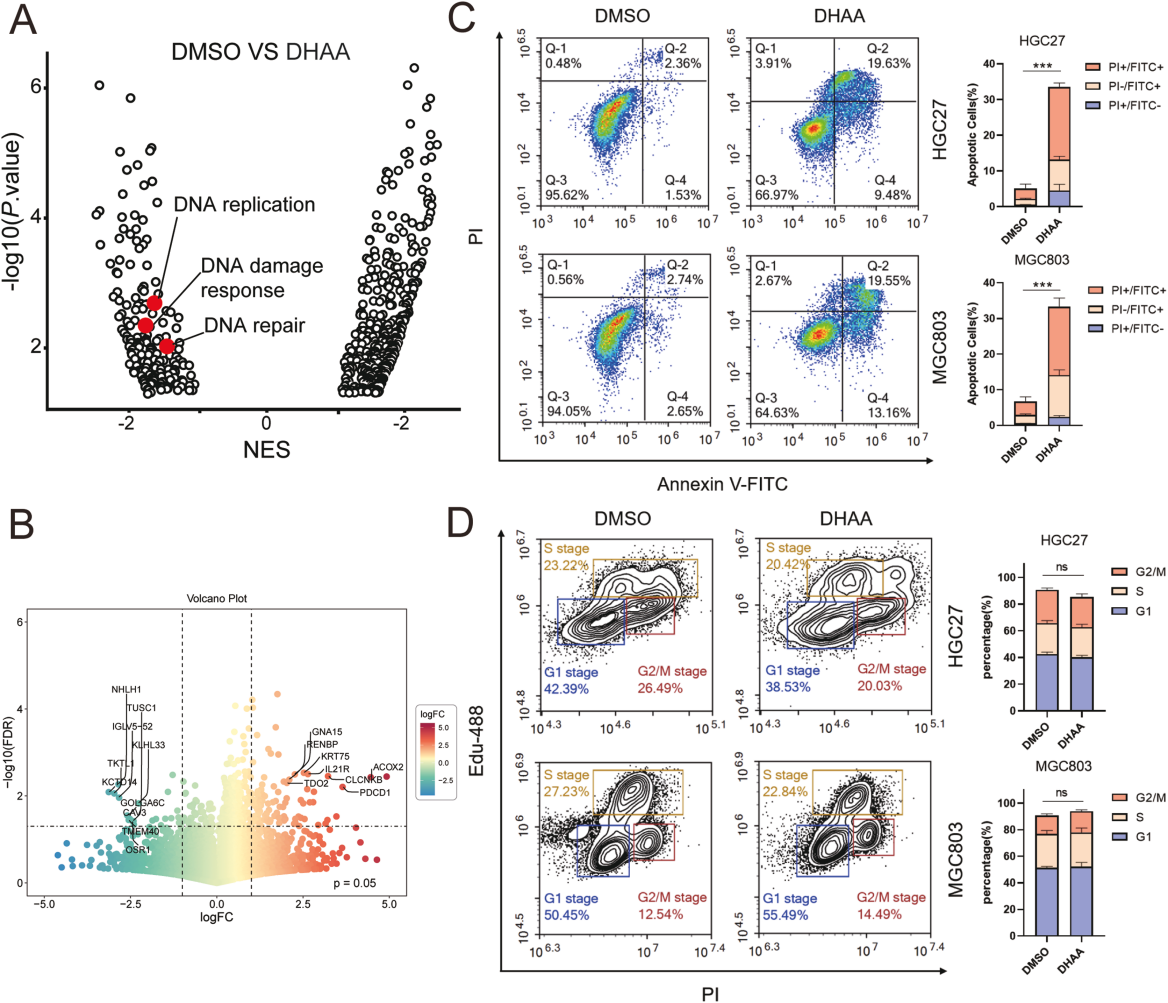
DHAA induces DNA damage and apoptosis in GC cells. (A) Normalized enrichment scores (NES) for all gene ontology sets differentially expressed in RNA sequencing of GC cells treated with DHAA versus DMSO. Gene ontology sets related to DNA damage are highlighted. (B) The differentially expressed genes after 4 μM DHAA treatment were presented as a Volcano plot. Genes with *P* < 0.01 and FC (fold change) > 2.0 were regarded as the candidate genes that showed a significant decrease or increase in RNA levels. (C) Cells were incubated with DHAA or DMSO for 24 h, followed by co-staining with propidium iodide (PI) and Annexin V-FITC, and then analyzed using flow cytometry. The proportion of PI^+^ Annexin V^+^, PI^+^ Annexin V^−^, and PI^−^ Annexin V^+^ were quantified and shown on the right side of this panel. (D) The cell cycle distribution of HGC27 and MKN45 cells after the treatment with DHAA or DMSO for 24 h. The distribution ratio of G1, S, and G2/M in the cell lines was determined and shown on the right side of this panel.

Gene expression differences treated with DHAA or DMSO were evaluated. Gene sets related to DNA replication, DNA damage response, and DNA repair were downregulated in the control group compared to those associated with unrelated cellular processes (Fig. 3A and B). Nucleic acid is the material basis of genes [30]. These results suggested that DHAA inhibited GC growth by inducing genomic imbalance, and apoptosis occurs in cells with ineffective or incomplete DNA repair [31, 32]. However, it was unknown that whether the DNA damage response induced by DHAA could induce apoptosis or not. Flow cytometry using the PI/Annexin V-FITC double-labeling analysis showed that DHAA-treated GC cells presented with an approximate rate of 30% apoptosis (Fig. 3C). We note that cell cycle-related signaling pathways are also enriched to some extent in sequencing results. HGC27 and MKN45 cells were treated with DHAA (4 μM) or DMSO for 24 h, and the cell cycle was analyzed by a flow cytometer. Our results showed that the cell cycle is not affected by DHAA. In other words, DHAA does not cause cell cycle arrest as thought (Fig. 3D).

### DHAA inhibits the expression of key enzymes in nucleotide metabolism in GC cells

**Figure 4:**
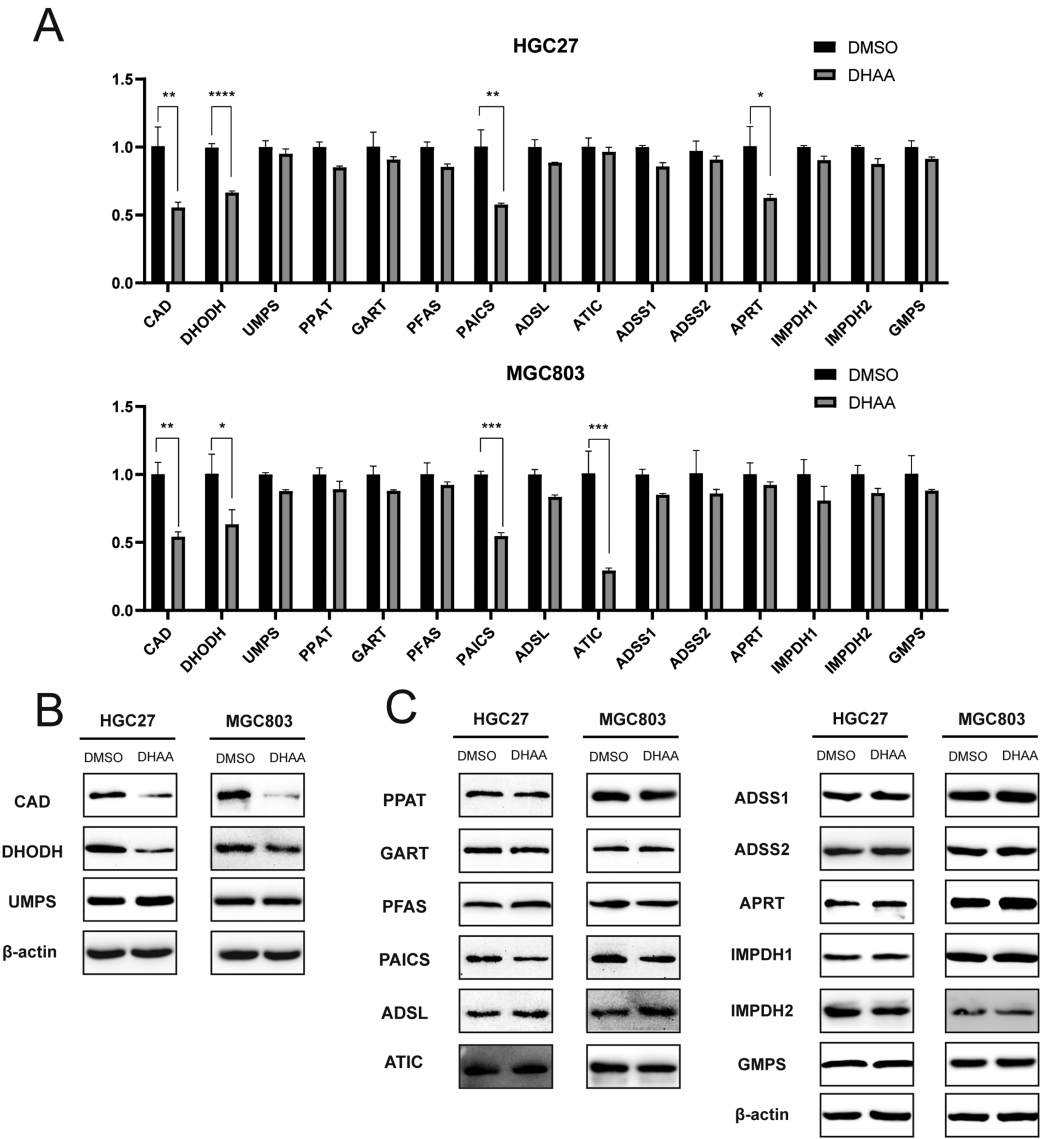
DHAA inhibits the expression of several key enzymes in nucleotide metabolism in GC cells. (A) The RNA levels of key enzymes in pyrimidine metabolism (CAD, DHODH, and UMPS) and purine metabolism (PPAT, GART, PFAS, PAICS, ADSL, ADSS1, ADSS2, APRT, IMPDH1, IMPDH2, and GMPS). (B, C) The protein levels of key enzymes in pyrimidine metabolism (B) and purine metabolism (C).

Nucleotide metabolism is a multistep process involving a series of catalytic enzymes, including lyases, synthases, amidotransferases, dehydrogenases, and so on. Among them, PPAT, phosphoribosylformylglycinamidine synthase (PFAS), ATIC, IMPDH, adenylosuccinate lyase (ADSL), dihydroorotate dehydrogenase (DHODH), and other rate-limiting enzymes are the most important factors in regulating nucleotide metabolism [7]. Next, we carried out qRT-PCR to determine whether the expression levels of enzymes involved in nucleotide metabolic pathways were changed after DHAA treatment. Consistent with the RNA sequencing results, all key enzymes in nucleotide metabolism tended to be downregulated (Fig. 4A). Notably, CAD, adenine phosphoribosyltransferase (APRT), phosphoribosylaminoimidazole carboxylase (PAICS), and ATIC levels were significantly reduced in MGC-803 cells after DHAA treatment, and CAD, APRT, PAICS, and DHODH levels were significantly reduced in HGC-27 cells after DHAA treatment (Fig. 4A). Western blotting proved that CAD and DHPDH were the only enzymes involved in pyrimidine metabolism that were downregulated and that PAICS was the only enzyme involved in purine metabolism that was downregulated in the DHAA-treated group compared to the control group (Fig. 4B and C).

These data suggest that DHAA affects nucleotide metabolism in GC cells, with the key pyrimidine metabolism-related enzymes CAD and DHODH and the key purine metabolism-related enzyme PAICS being significantly downregulated.

### DHAA induces apoptosis in GC cells by reducing the expression of transcription factor FOXK2

**Figure 5:**
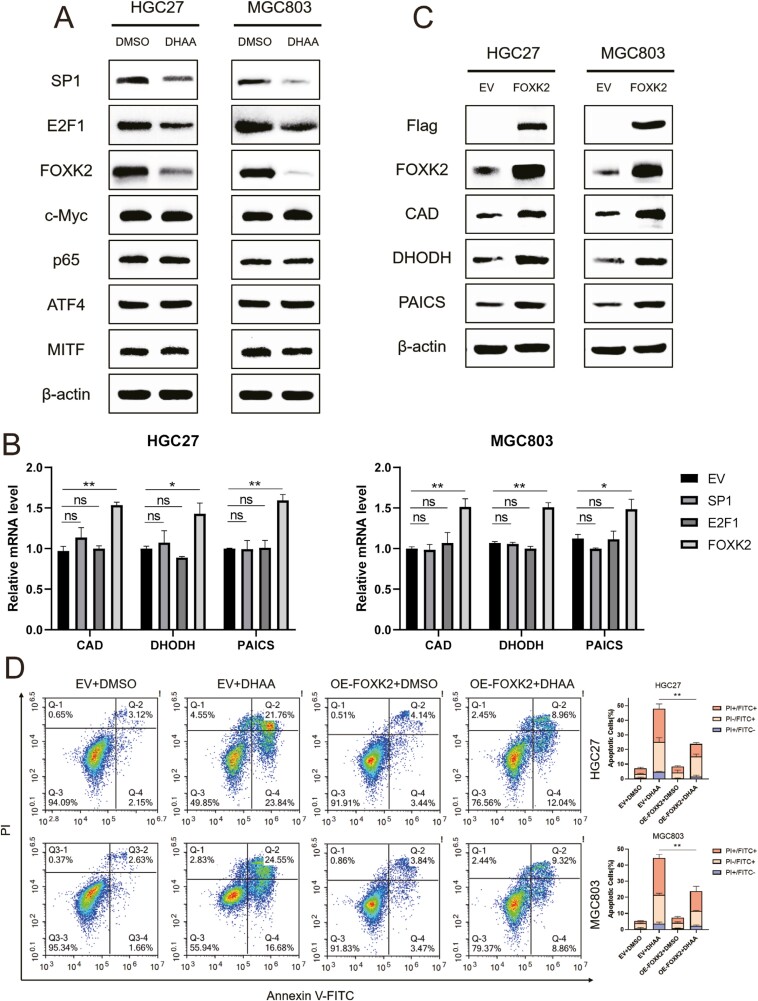
DHAA induces apoptosis in GC cells by reducing the expression of transcription factor FOXK2. (A) Immunoblotting analysis of the indicated proteins in HGC27 and MKN45 cells were treated with DHAA (4 μM) for 24 h and with anti-β-actin antibody as a loading control. (B) qRT-PCR was performed in HGC27 and MKN45 cells to determine the mRNA level of CAD, DHODH, and PAICS. Data are presented as mean ± SEM. Student’s *t*-test, *P < .05, **P < .01. (C) HGC27 and MKN45 cells overexpressing Flag-FOXK2 were treated with DHAA (4 μM) for 48 h. The efficiency of overexpression was detected by the expression levels of Flag and FOXK2. CAD, DHODH, PAICS, and β-actin expression were detected. Immunoblotting analyses were performed as indicated. (D) Cells expressing empty vector (EV) or FOXK2 were incubated with DHAA or DMSO for 48 h, followed by co-staining with propidium iodide (PI) and Annexin V-FITC, and then analyzed using flow cytometry. The proportion of PI^+^ Annexin V^+^, PI^+^ Annexin V^−^, and PI^−^ Annexin V^+^ were quantified and shown on the right side of this panel.

The maintenance of intracellular nucleotide levels is crucial for cell survival and fate [33]. *De novo* nucleotide metabolic enzymes are strictly regulated at the transcriptional level. Previous reports have demonstrated that multiple key genes for nucleotide biosynthesis can be directly regulated by a single transcription factor to promote nucleotide biosynthesis and tumor growth [17, 34, 35]. To further elucidate the potential mechanism of DHAA in GC cells, we examined the expression levels of several transcription factors that promote transcription of nucleotide metabolism genes and found that Sp1 transcription factor (SP1), E2F transcription factor 1 (E2F1), and Forkhead box K2 (FOXK2) were significantly reduced by DHAA treatment (Fig. 5A). What calls for special attention is that Li *et al*. [36] reported that FOXK2 binding sites at the promoter regions of the nucleotide synthetic genes (CAD, DHODH, PAICS, etc), and mutation of the binding motifs abolished FOXK2 binding to its target genes and the subsequent transcriptional roles of FOXK2 on its target gene. We also demonstrated that CAD, DHODH, and PAICS were significantly increased at both transcriptional and protein levels in FOXK2 overexpressing GC cells, but not in SP1 or E2F1 overexpressing GC cells (Fig. 5B and C and Supplementary Fig. 2A and B). FOXK2 belongs to the fork head box (FOX) transcription factor superfamily, which plays important roles in cell proliferation, differentiation, autophagy, and aerobic glycolysis [37, 38]. The forkhead/winged-helix motif (GTAAACA) is the FOXK2 binding site in the genome [39]. DHAA significantly restricted FOXK2, thereby reducing the expression of nucleotide-metabolizing enzymes and interfering with normal biosynthesis. Combining our results with the previous research, does this mean that FOXK2 is involved in DHAA-induced apoptosis? To test this hypothesis, the percentage of apoptosis in cells overexpressing FOXK2 under DHAA treatment was detected through flow cytometry, which showed a significant decrease in the number of apoptotic cells, with a decrease of approximately half compared to the control group (Fig. 5D). Unexpectedly, the expression of SP1 and E2F1 did not reverse the occurrence of DHAA mediated apoptosis (Supplementary Fig. 2C and D). The current results suggested that DHAA aims to limit tumor growth by interfering with the transcription of key nucleotide metabolism genes by FOXK2.

To determine whether DHAA and FOXK2 interact directly, we performed dock studies using the FOXK2 protein structure predicted based on Alphafold2. The results indicated that the central moiety of DHAA is located at the ligand-binding pocket and the binding energy between FOXK2 protein and DHAA is −7.5 kcal/mol. Protein-Ligand Interaction Profiler (PLIP) analysis showed that DHAA formed hydrogen bonds with PHE74, TYR295, and ALA298 of FOXK2 protein sequence, with lengths of 3.54Å, 3.90Å, and 3.86Å, respectively. The schematic illustration for the binding between DHAA and FOXK2 shows a large number of hydrophobic groups formed near the binding site, and the formation of such hydrophobic cores increases protein instability (Supplementary Fig. 2E–G). From the above data, it can be inferred that DHAA can interfere with the transcription and expression of key genes in the nucleotide metabolism pathway by interfering with transcription factor FOXK2 and restricting nucleotide metabolism to affect the growth of GC cells.

### DHAA exerts antitumor effects *in vivo*

**Figure 6:**
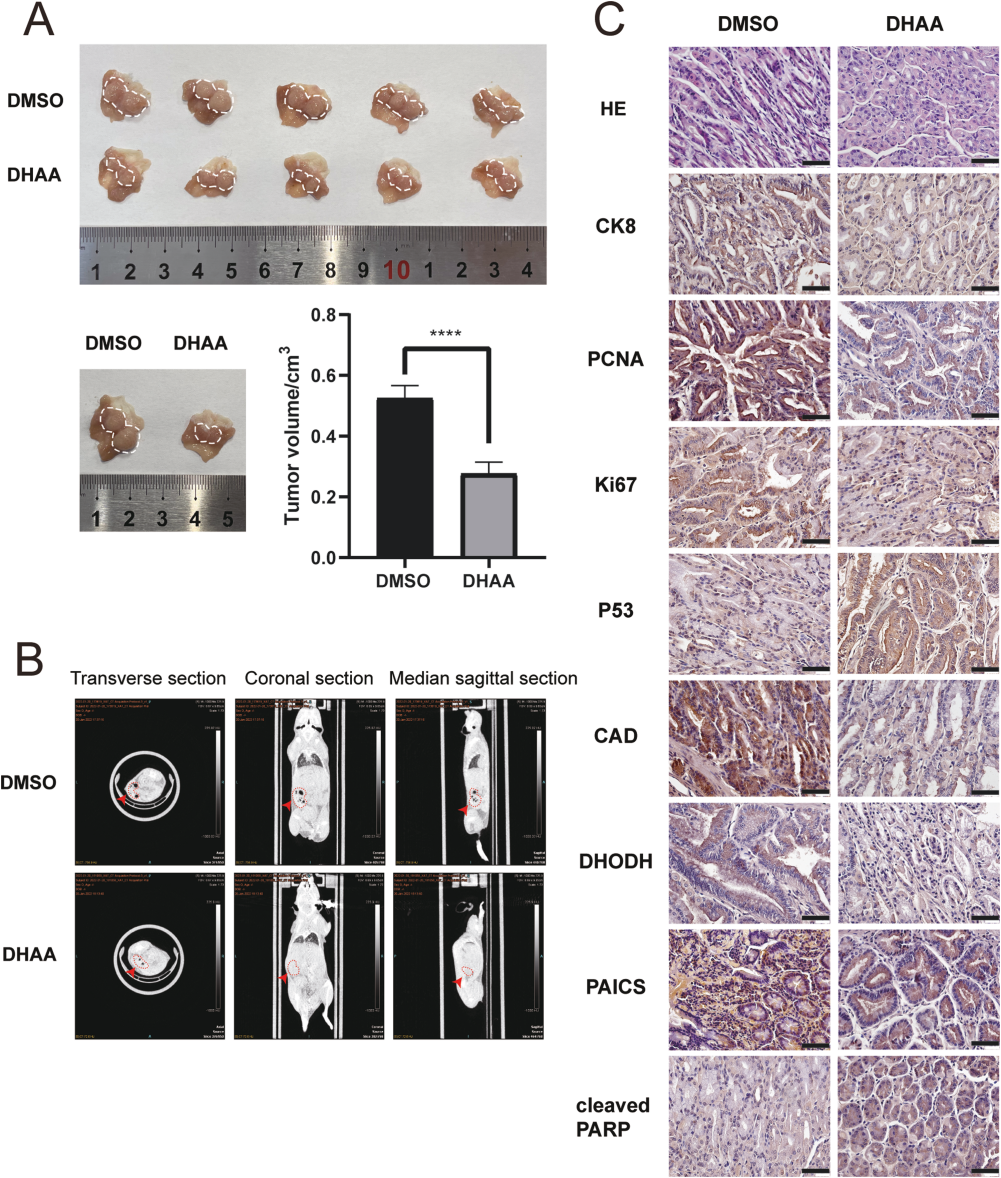
Effects of DHAA on tumor growth in *K19-Wnt1/C2mE* transgenic mice. (A) Images of tumors in *K19-Wnt1/C2mE* transgenic mice after different treatments. Tumor volumes were compared to assess the efficacy of DHAA treatment. (B) The indicated mice were subjected to micro-CT. (C) HE staining was performed, and the expression levels of CK8, PCNA, Ki67, p53, CAD, DHODH, PAICS, and cleaved PRARP were measured by IHC staining (scale bars, 25 μm).

To evaluate the therapeutic effect of DHAA *in vivo*, *K19-Wnt1/C2mE* transgenic mice were employed. According to previous reports [26, 40], metaplasia, hyperplasia, and tumor growth occur in the glandular stomach of these transgenic mice. According to previous reports, 28-week-old mice were selected for follow-up experiments. They were randomly split into two groups: the control group and the DHAA group (7.5 mg/kg). It was found that tumors in the control group grew rapidly, while DHAA treatment significantly inhibited tumor growth (Fig. 6A and B).

Histological observation is an important method for evaluating the progression of GC. H&E staining showed that the gland arrangement in the DHAA-treated group was more orderly than that in the untreated group, suggesting that DHAA significantly inhibited GC progression. Next, we measured the protein levels of Ki-67, cytokeratin 8 (CK8), proliferating cell nuclear antigen (PCNA), p53, cleaved PARP, CAD, DHODH, and PAICS by immunohistochemical (IHC) staining (Fig. 6C). As shown in Fig. 6C, the levels of the oncogenes Ki-67, CK8, and PCNA were markedly decreased, and the expression of the tumor suppressor gene p53 and the apoptosis marker cleaved PARP was obviously upregulated after DHAA treatment, indicating that DHAA could restrain the growth of GC by inducing apoptosis. Moreover, the protein expression levels of CAD, DHODH, and PAICS in DHAA-treated mice were lower, indicating that nucleotide metabolism was inhibited, which was consistent with the results of the *in vitro* experiments. Collectively, these findings suggest that DHAA exerts its anticancer effect on GC by reducing the expression of CAD, DHODH, and PAICS, which are involved in nucleotide metabolism.

## Discussion

Previous studies have shown that DHAA has certain anticancer activity, but the effect of DHAA on GC remains unclear. In this research, we explored the effects of DHAA on GC cells both *in vitro* and *in vivo*. The results demonstrated that DHAA could reduce the viability and inhibit the proliferation of GC cells by disrupting nucleotide metabolism.

Classic tumor therapies aim to promote tumor cell apoptosis, but many therapeutic strategies are limited by drug resistance or are ineffective in inducing tumor cell apoptosis, and new solutions are urgently needed [41]. Metabolic pathways in tumor cells can be reorganized to promote tumor progression metastasis and chemotherapy resistance, which is one of the most critical factors leading to cancer treatment failure [42]. For example, many studies have reported that increased glucose uptake and aerobic glycolysis by cancer cells promote uncontrolled cell proliferation [43, 44]. During cancer development, in addition to glucose metabolism being abnormally regulated, nucleotide metabolism is greatly disrupted [45–47].

In this research, we report for the first time that DHAA reduces viability and inhibits the proliferation of two types of GC cells by disrupting nucleotide metabolism. Initially, we discovered that DHAA has a significant inhibitory effect on GC cell and organoid proliferation. To explore the underlying mechanism, we conducted RNA sequencing. The RNA sequencing showed that the activity of the purine metabolism pathway and pyrimidine metabolism pathway was apparently decreased, and the activity of pathways related to cell proliferation, such as DNA replication, DNA repair, the RNA polymerase pathway, and cell cycle-related pathways, was also decreased. Based on this, we speculated that DHAA may inhibit cell proliferation by affecting the nucleotide metabolic pathway, thus exerting an anticancer effect. To verify our speculation, we used Western blotting and qRT-PCR to verify changes in the expression of several key enzymes in nucleotide metabolism after DHAA treatment. As we expected, the expression levels of these key enzymes showed a downward trend, along with the levels of CAD, DHODH, and PAICS being significantly decreased. DHAA inhibits proliferation by targeting the decrease of FOXK2, a transcription factor that directly regulates the expression of nucleotide synthetic genes. We also found that in a mouse model, DHAA could delay tumorigenesis and reduce the levels of CAD, DHODH, and PAICS.

CAD and DHODH exhibit pivotal enzymatic activities to contribute to the *de novo* synthesis of pyrimidine nucleotides in all living organisms, and CAD and DHODH levels are closely related to pyrimidine levels in the body [48]. As a trifunctional enzyme, CAD synthesizes dihydroorotic acid from CO_2_ and glutamine, the raw materials of pyrimidine metabolism, through three steps. DHODH further converts dihydroorotic acid to orotate, a key product in the pyrimidine synthesis pathway. In tumor cells, a strong pyrimidine flux is essential to satisfy additional demands for nucleic acids and other cellular constituents [49, 50]. Elevation of the levels of CAD and DHODH, which catalyze the initial step of pyrimidine synthesis, has been reported to directly increase nucleotide levels in several cancers, affecting cancer prognosis [49, 51–54]. The PAICS gene encodes a bifunctional enzyme with phosphoribosamine amidazole carboxylase activity at the N-terminus and PAICS activity at the C-terminus, catalyzing the formation of *N*-succinyl-5ʹ-imidazole-4ʹ-formamide nucleotide (SAICAR) from 5ʹ-imidazole-4ʹ-formamide nucleotide (AIR) [55]. Additionally, PAICS can bind proteins [56]. Previous studies have demonstrated that PAICS plays a significant role in some tumors and is related to tumor proliferation and metastasis [57–59].

DHAA has recently attracted widespread interest owing to its potent anticancer properties and has been successfully formulated as a nanoparticle-based liposomal drug delivery system [60]. The mechanistic understanding of the antitumor effect of DHAA is restricted to a few publications using melanoma cell lines. The antitumor effect of DHAA in melanoma cell lines *in vitro* and *in vivo* was associated with the inhibition of Akt, Stat3, and Erk1/2 activation (reduced phosphorylation). The inhibition of these pro-survival and oncogenic pathways upon DHAA treatment was observed as early as 3 h after treatment at 3–5 μM concentrations [22]. However, the precise role and contribution of these pathways in growth inhibition and cell death induction by DHAA is still unclear. Furthermore, the direct interaction of DHAA reduces the expression of FOXK2. Whether DHAA can make target proteins more easily recognized and degraded by ubiquitin-proteasome or autophagy-lysosome systems needs further exploration [61]. This study reveals DHAA’s targeted nucleotide metabolism and, based on the metabolic vulnerability it causes, we believe that by modifying DHAA’s structure to optimize its biological activity, it can be more effective in treating specific diseases. For example, by introducing specific functional groups or changing the steric configuration of DHAA, the binding affinity of compounds to potential target proteins can be increased, thereby enhancing their pharmacological effects.

## Conclusion

In conclusion, our study suggests that DHAA is a drug similar to 5-FU that regulates nucleotide metabolism and has the potential to inhibit cell viability and proliferation. Our data support the use of DHAA as a possible therapeutic compound for GC. Further research and large-scale, multicenter collaborative clinical trials are needed in the future to explore the mechanism underlying the effect of DHAA and its potential for clinical application.

## Supplementary data

Supplementary data is available at *Carcinogenesis* online.

**Supplementary Figure 1** Anticancer activity of DHAA in GC organoids. (A) Chemical structures of DHAA. (B) Human GC organoid was seeded at ~100 crypts/well and treated with various concentrations of DHAA for 24 h. At the end of the incubation period, organoid viability was assessed using CellTiter-Glo® 3D, and treated wells were compared to solvent control wells to generate dose-response curves. IC_50_ value was compared. (C, D) GO functional enrichment analysis of DNA damage-related pathways. (E, F) GO functional enrichment analysis of nucleotide metabolism-related pathways. (G) Gene set enrichment analysis of DNA replication, DNA damage response, and DNA repair pathways in GC cells treated with DHAA versus DMSO. NES = normalized enrichment score. (H) KEGG pathway analysis revealed that the differentially expressed genes were enriched in the nucleotide metabolic pathway and DNA replication in GC organoids. (I) Analysis of the relationships between pathways associated with the differentially expressed genes in GC organoids.

**Supplementary Figure 2** FOXK2 transcriptionally regulates nucleotide metabolism enzymes, rather than SP1 and E2F1. (A) HGC27 and MKN45 cells overexpressing Flag-SP1 were treated with DHAA (4 μM) for 48 h. The efficiency of overexpression was detected by the expression levels of Flag and SP1. CAD, DHODH, PAICS, and β-actin expression were detected. (B) HGC27 and MKN45 cells overexpressing Flag-E2F1 were treated with DHAA (4 μM) for 48 h. The efficiency of overexpression was detected by the expression levels of Flag and E2F1. CAD, DHODH, PAICS, and β-actin expression were detected. Immunoblotting analyses were performed as indicated. (C) Cells expressing empty vector (EV) or SP1 were incubated with DHAA or DMSO for 48 h, followed by co-staining with propidium iodide (PI) and Annexin V-FITC, and then analyzed using flow cytometry. (D) Cells expressing empty vector (EV) or E2F1 were incubated with DHAA or DMSO for 48 h, followed by co-staining with propidium iodide (PI) and Annexin V-FITC, and then analyzed using flow cytometry. The proportion of PI^+^ Annexin V^+^, PI^+^ Annexin V^−^, and PI^−^ Annexin V^+^ were quantified and shown on the right side of this panel. (E) A direct interaction between DHAA and FOXK2. Computational molecular docking analysis to investigate the interaction of DHAA binding to FOXK2 (AlphaFold2). (F) The schematic illustration for the binding between FOXK2 and DHAA is presented by LigPlot. (G) Description of specific key information of 3D docking graph.

bgae037_suppl_Supplementary_Materials

## Data Availability

The sequencing data reported in this study are being uploaded to the Sequence Read Archive (SRA) with the submission number SUB11194014.
