# Supplementary material for: Dehydroabietylamine exerts antitumor effects by affecting nucleotide metabolism in gastric cancer
Source: Carcinogenesis. 2024 Jun 13;45(10):759–72. doi: 10.1093/carcin/bgae037 (PMC11464700; doi:10.1093/carcin/bgae037)

Raw data for Western Blots

Figure 4B

Raw data for Western Blots of CAD(240KDa)

HGC27
Up Lane 1: DMSO; Up Lane 2: DHAA

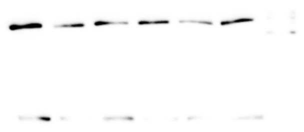

Lane 1: DMSO; Lane 2: DHAA

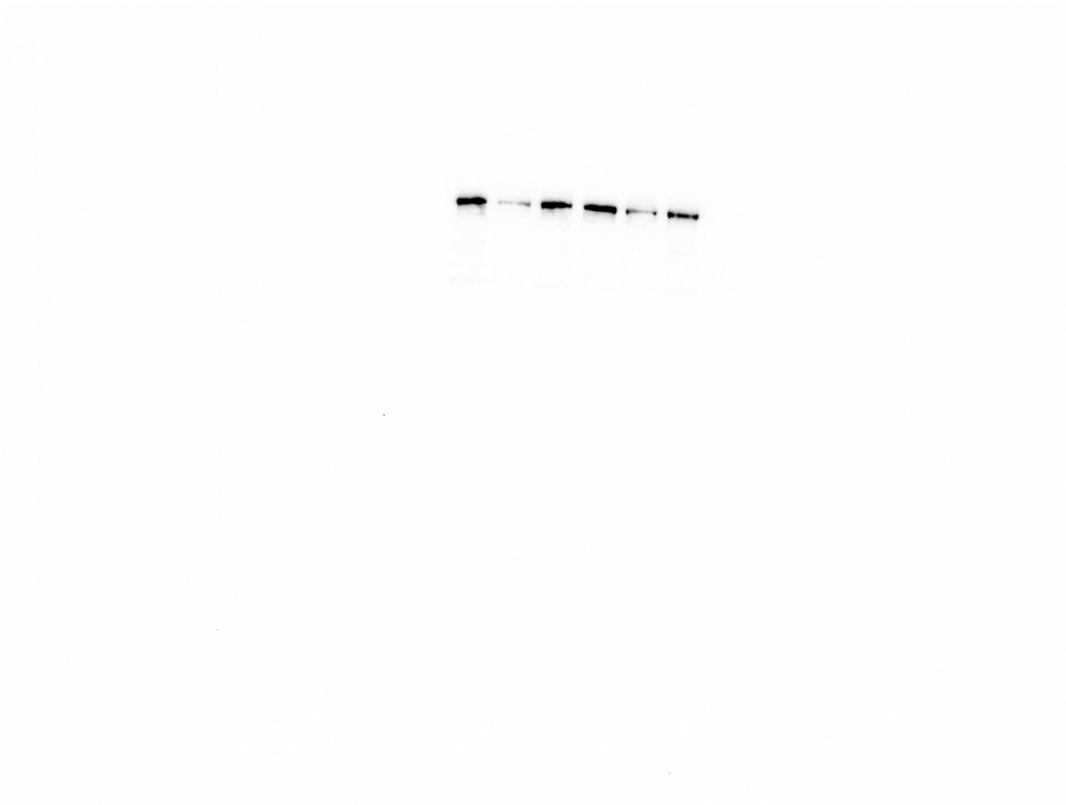

Raw data for Western Blots of DHODH(43KDa)

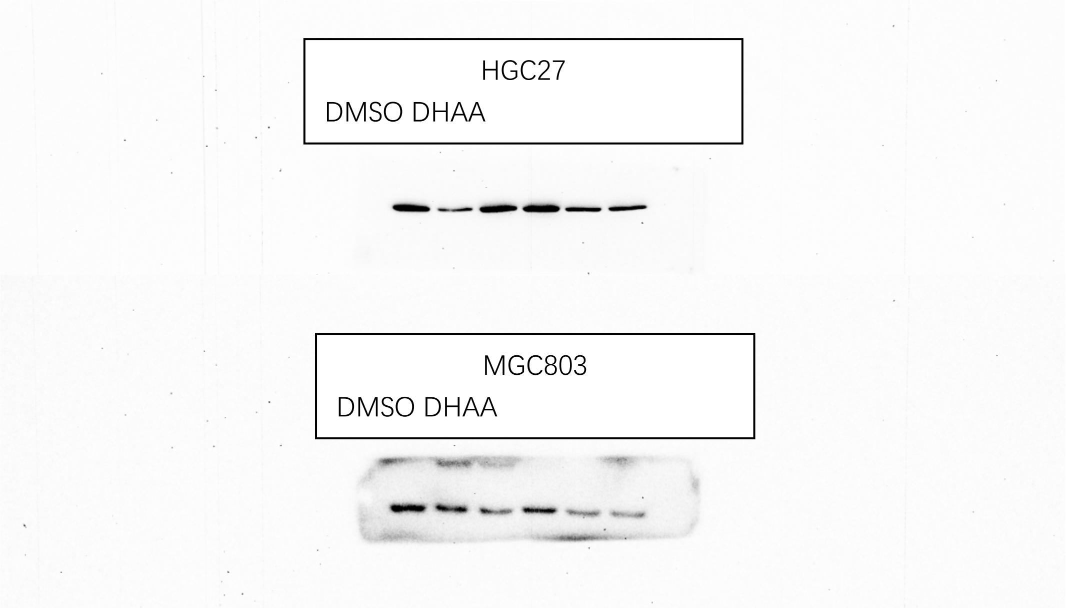

Raw data for Western Blots of UMPS(57KDa)

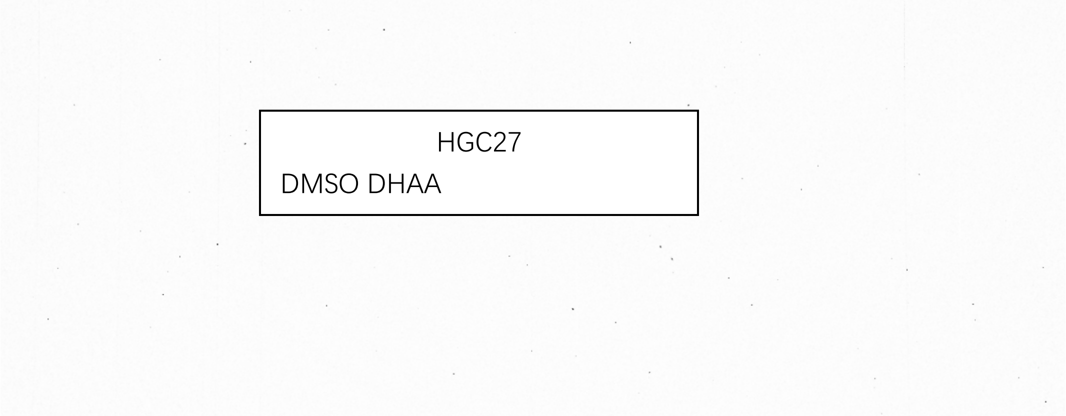

MGC803

DMSO DHAA

Raw data for Western Blots of β-actin(42KDa)

HGC27

DMSO DHAA

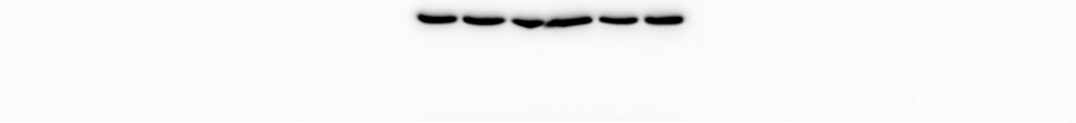

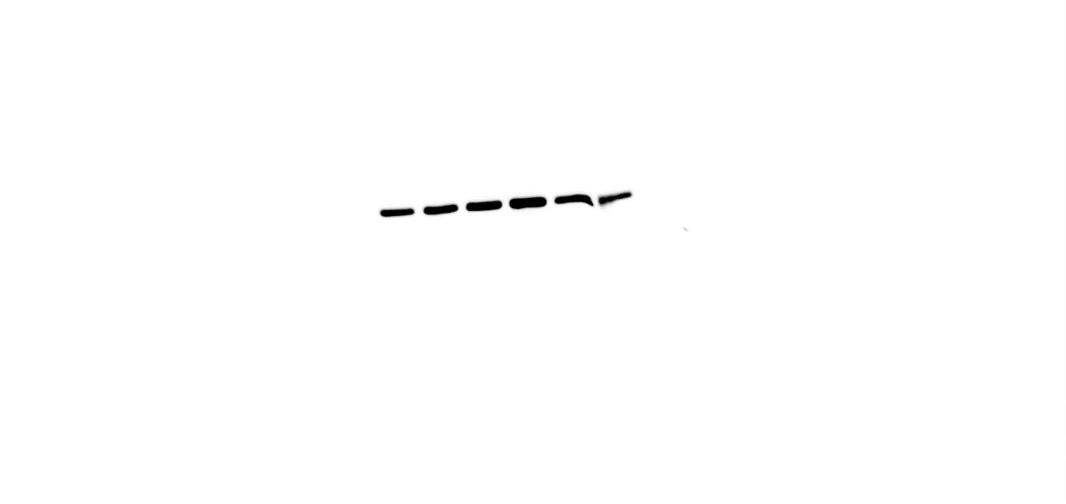

MGC803

DMSO DHAA

Figure 4C

Raw data for Western Blots of PPAT(55KDa)

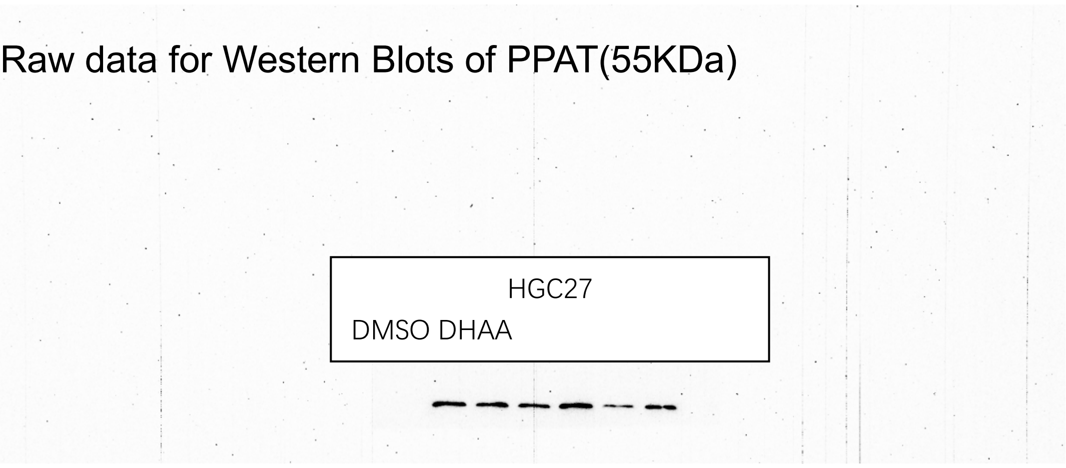

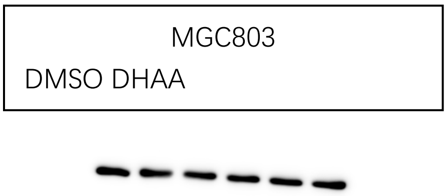

Raw data for Western Blots of GART(120KDa)

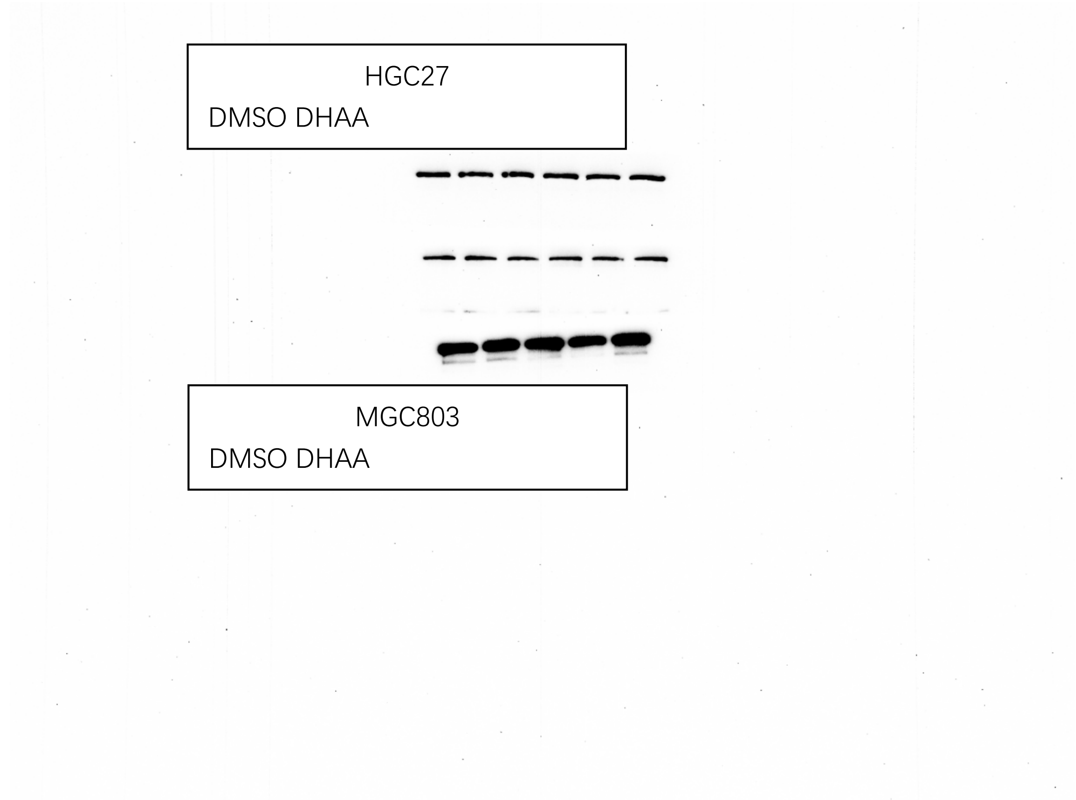

Raw data for Western Blots of PFAS(145KDa)

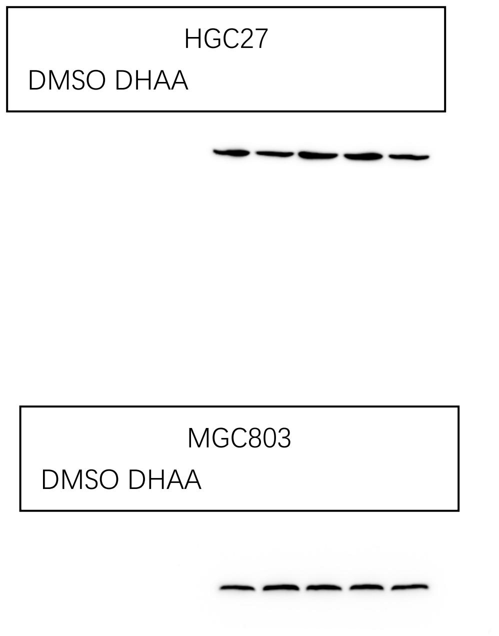

Raw data for Western Blots of PAICS(47KDa)

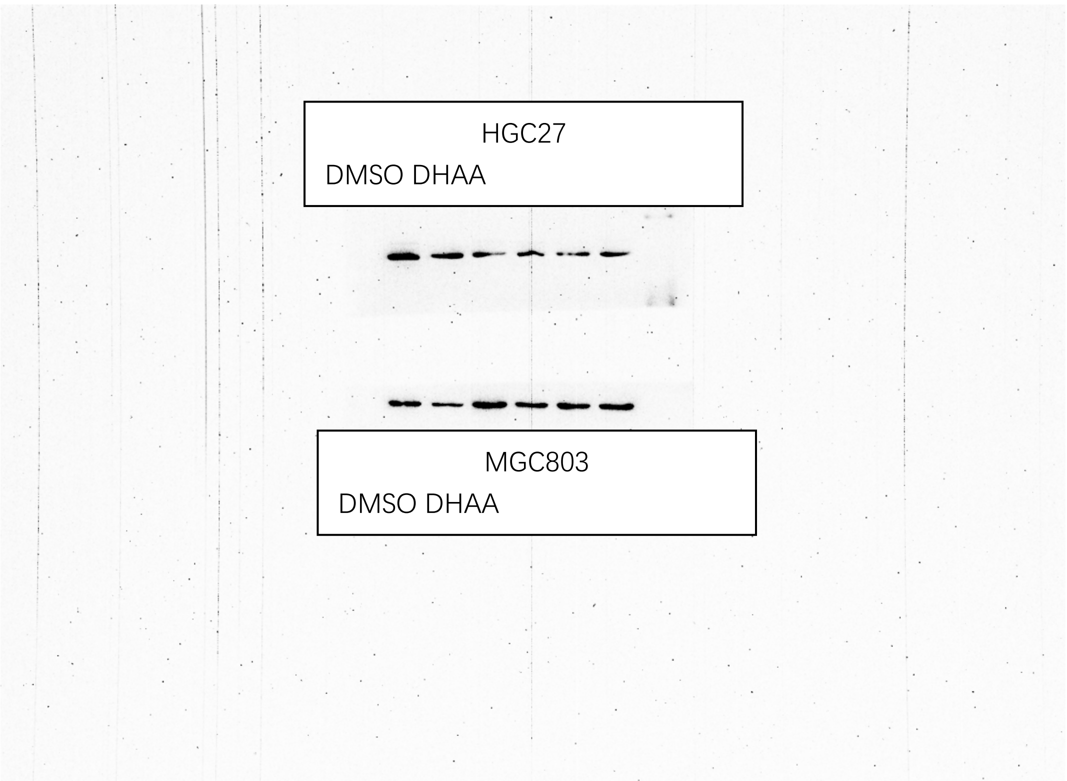

Raw data for Western Blots of ADSL(55KDa)

HGC27

DMSO DHAA

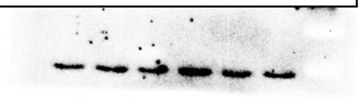

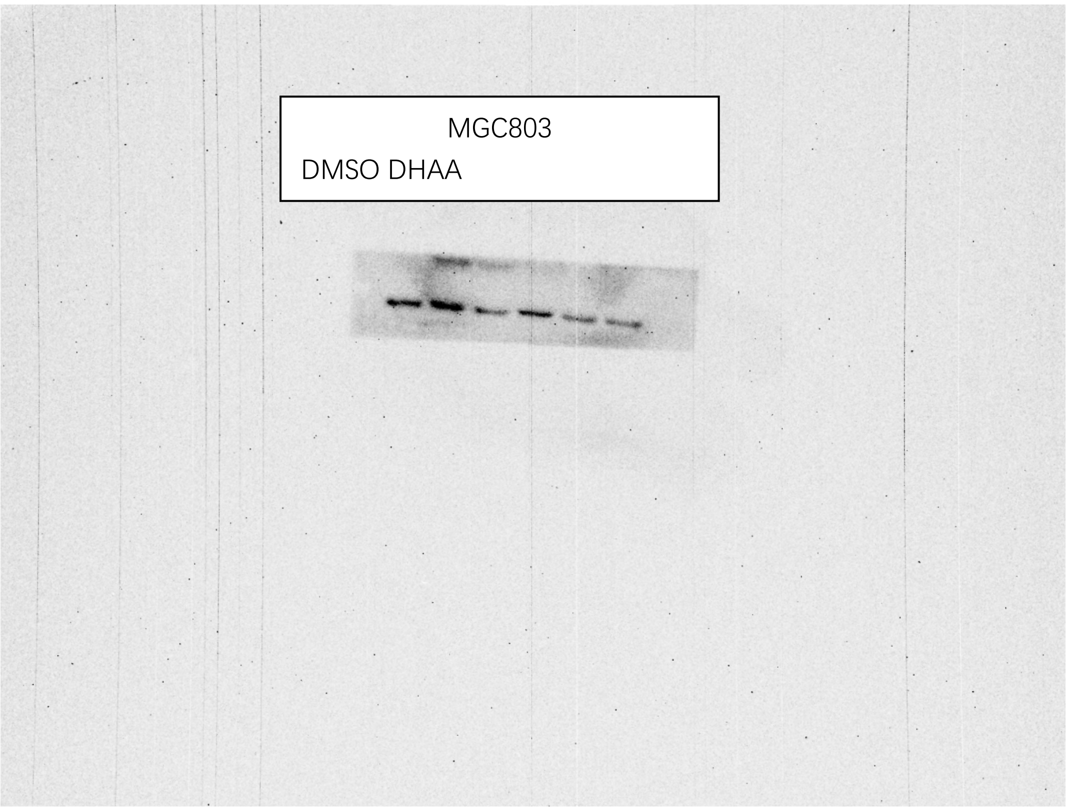

Raw data for Western Blots of ATIC(62KDa)

HGC27

DMSO DHAA

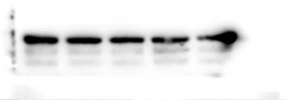

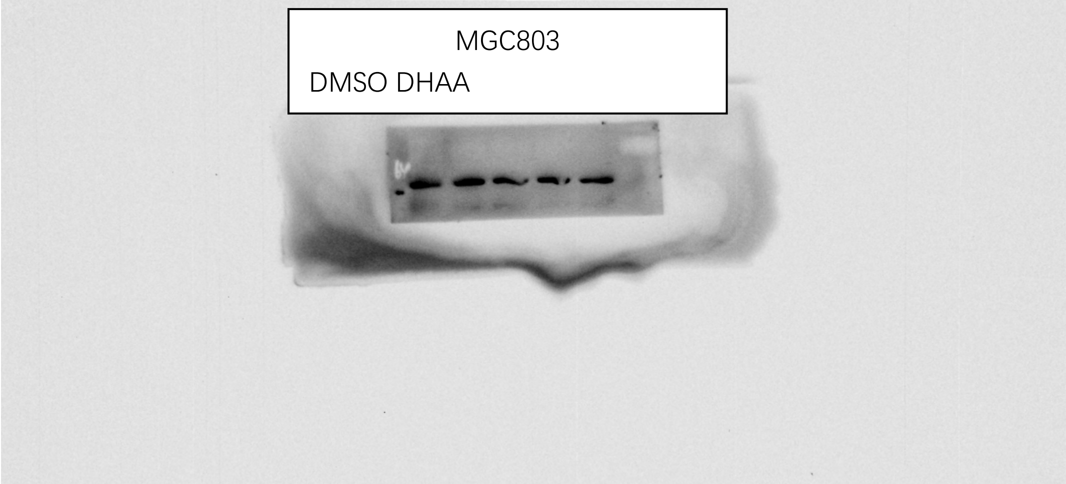

Raw data for Western Blots of ADSS1(52KDa)

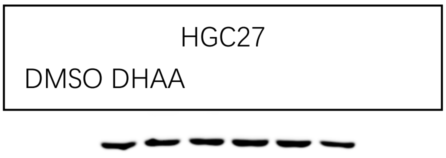

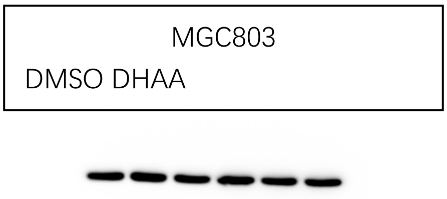

Raw data for Western Blots of ADSS2(52KDa)

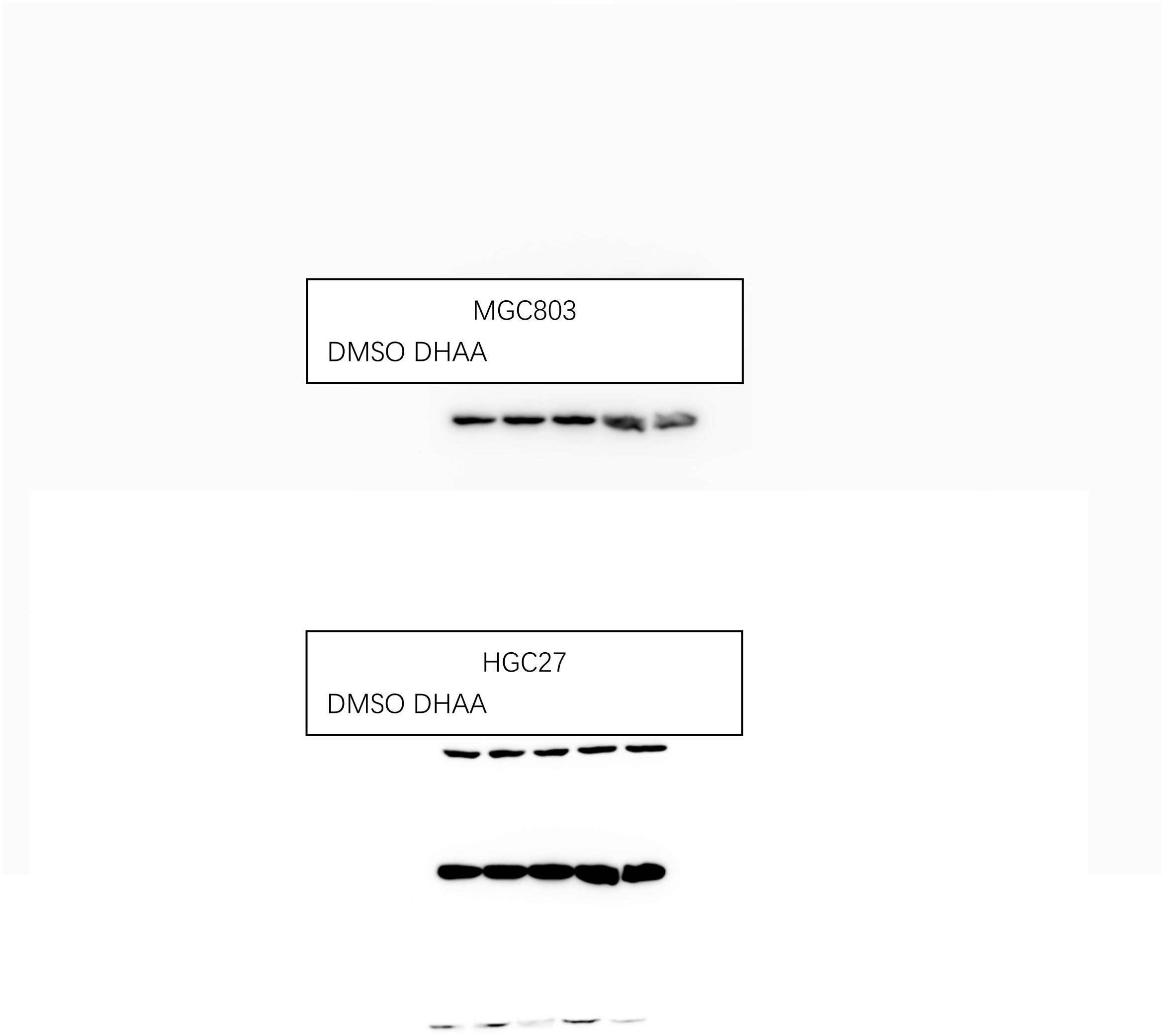

Raw data for Western Blots of APRT(20KDa)

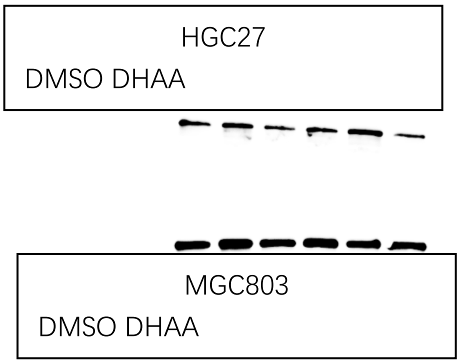

Raw data for Western Blots of IMPDH1(55KDa)

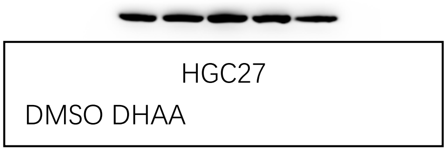

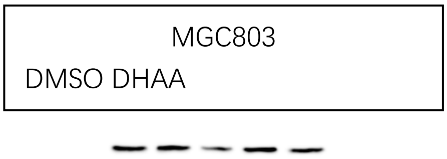

Raw data for Western Blots of IMPDH2(55KDa)

HGC27

DMSO DHAA

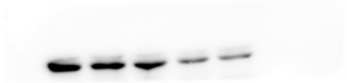

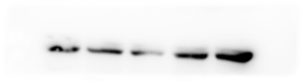

MGC803

DMSO DHAA

Raw data for Western Blots of GMPS(80KDa)

HGC27

DMSO DHAA

MGC803

DMSO DHAA

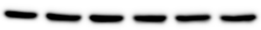

Raw data for Western Blots of β-actin(42KDa)

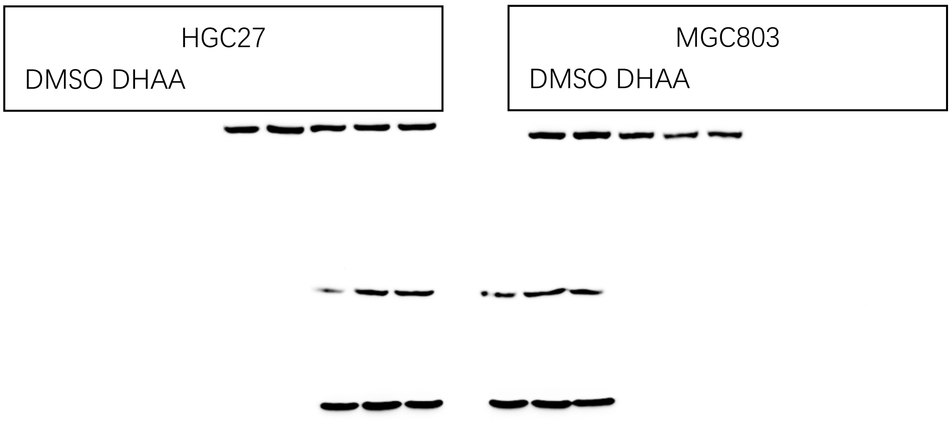

Figure 5A

Raw data for Western Blots of SP1(90KDa)

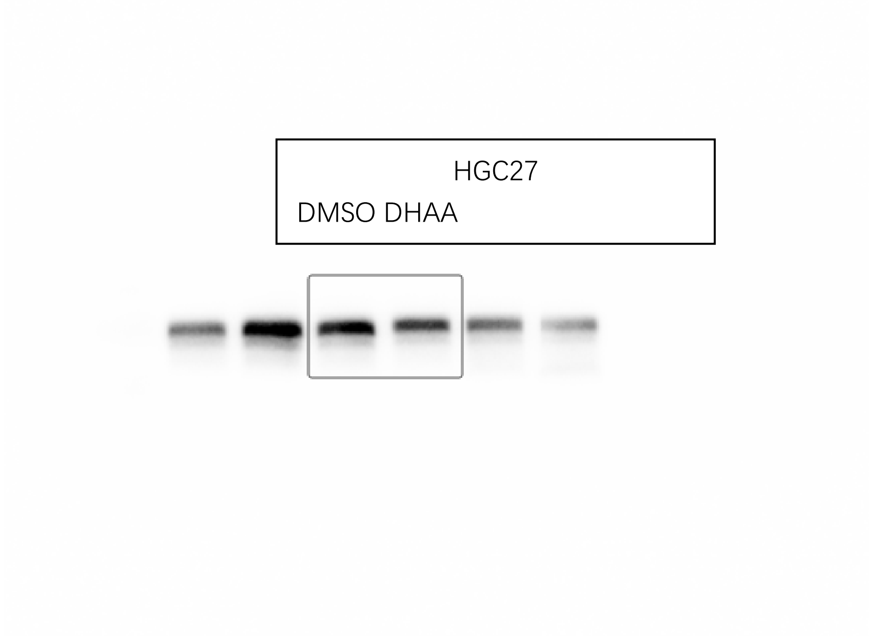

MGC803
DMSO DHAA

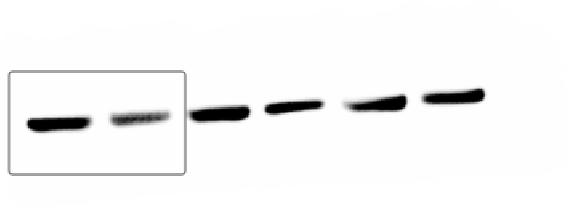

Raw data for Western Blots of c-Myc(60KDa)

HGC27
DMSO DHAA

MGC803
DMSO DHAA

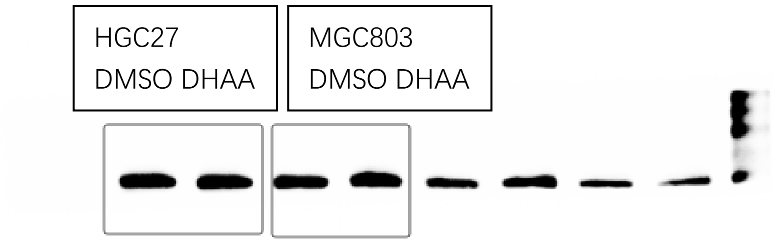

Raw data for Western Blots of p65(65KDa)

HGC27
DMSO DHAA

MGC803
DMSO DHAA

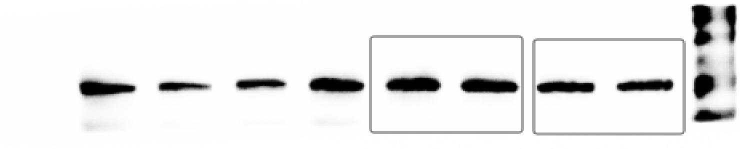

Raw data for Western Blots of ATF4(50KDa)

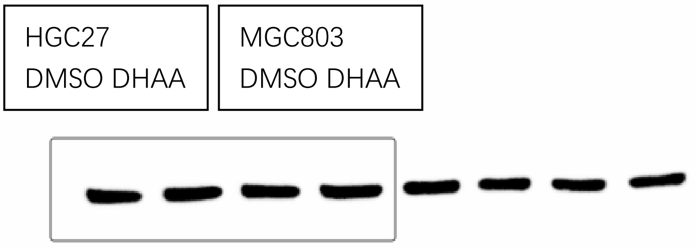

Raw data for Western Blots of MITF(59KDa)

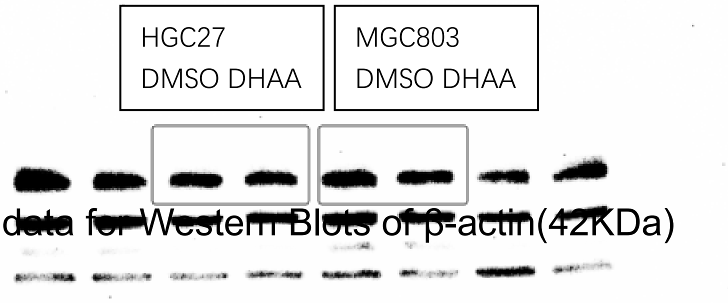

Raw data for Western Blots of β-actin(42KDa)

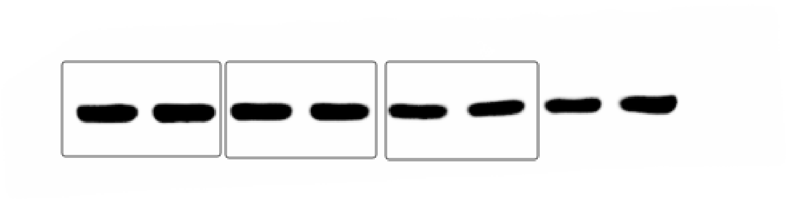

Figure 5C

Raw data for Western Blots of Flag-FOXK2(80KDa)

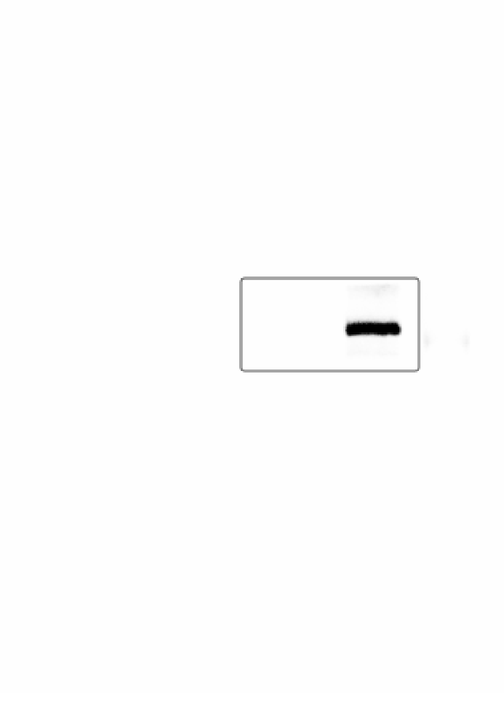

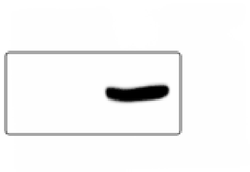

Raw data for Western Blots of E2F1(47KDa)

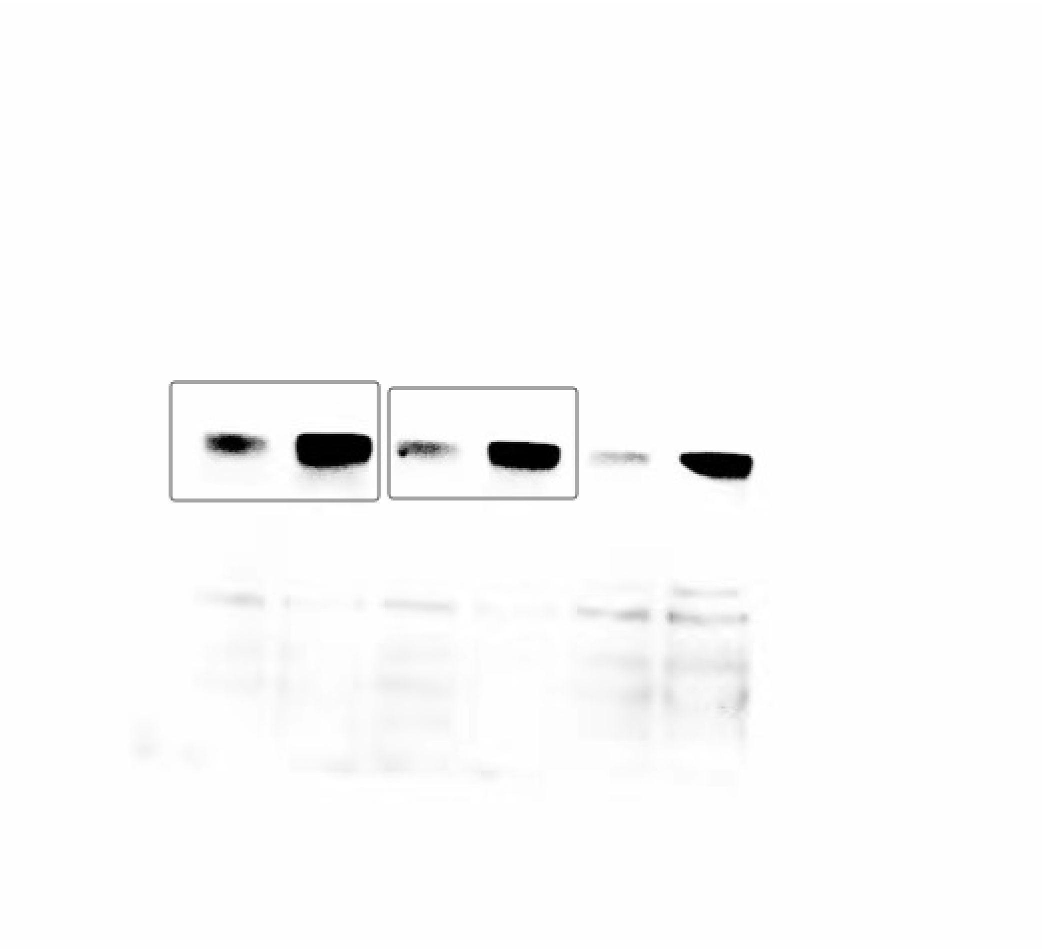

Raw data for Western Blots of CAD(240KDa)

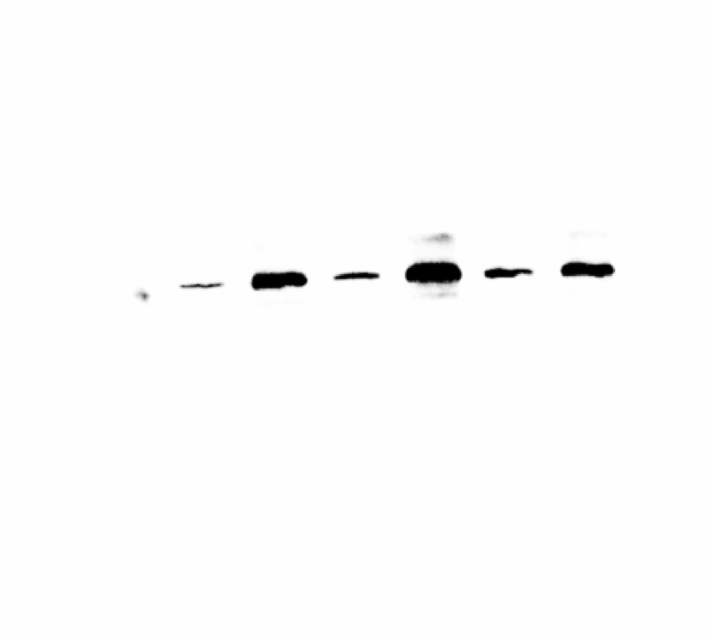

Raw data for Western Blots of DHODH(43KDa)

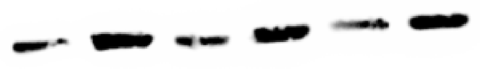

Raw data for Western Blots of PAICS(47KDa)

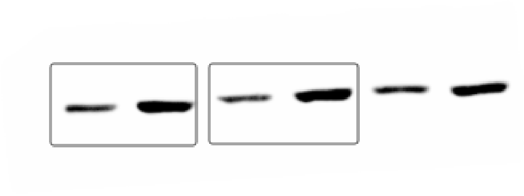

Supplementary Figure 2

Raw data for Western Blots of Flag-SP1(90KDa)

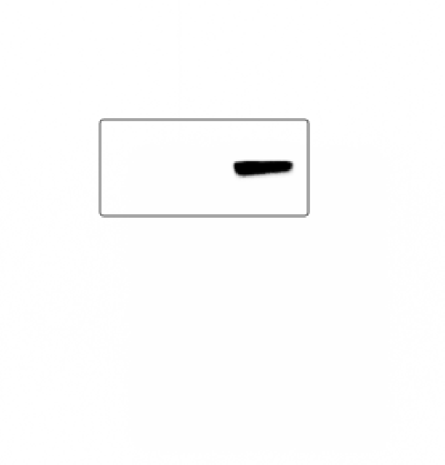

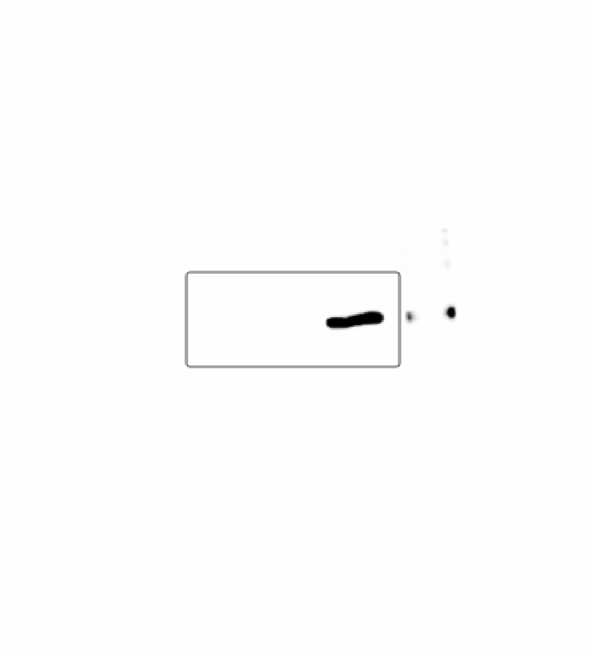

Raw data for Western Blots of Flag-E2F1(47KDa)

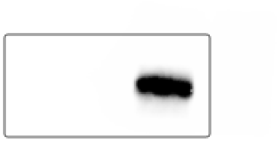

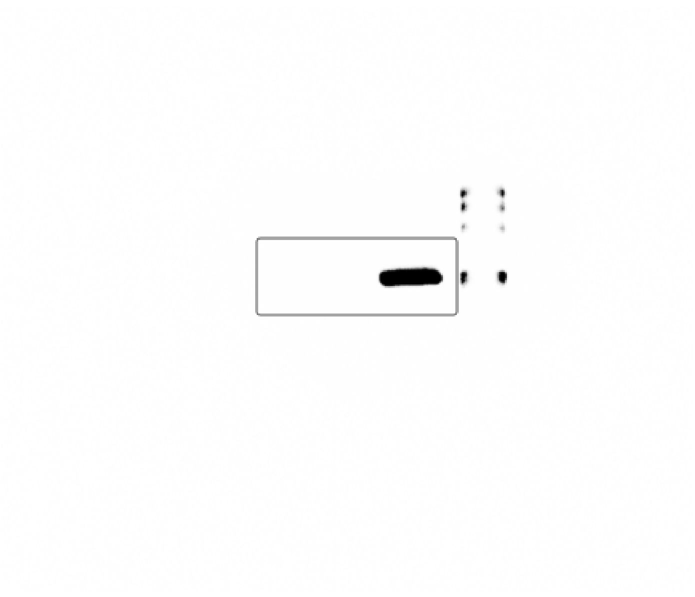

Raw data for Western Blots of SP1(90KDa)

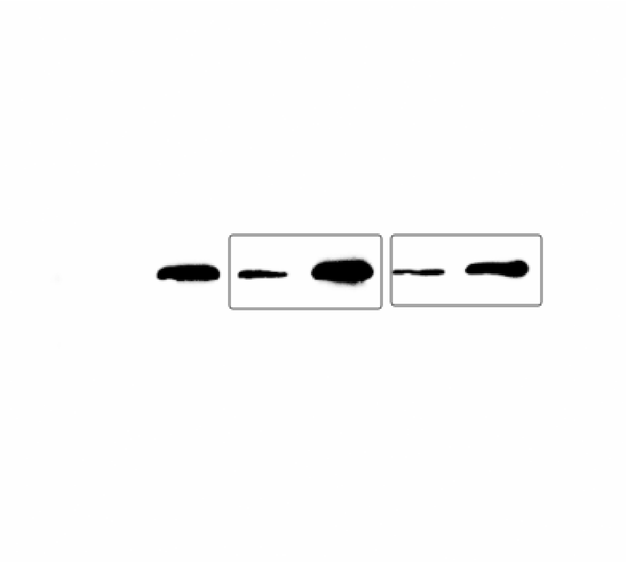

Raw data for Western Blots of E2F1(47KDa)

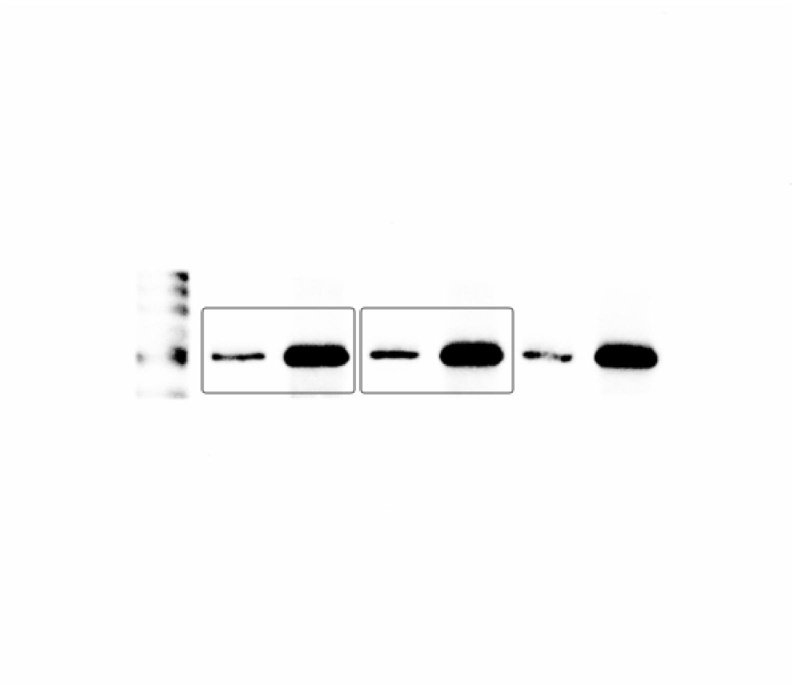

Raw data for Western Blots of CAD(240KDa)

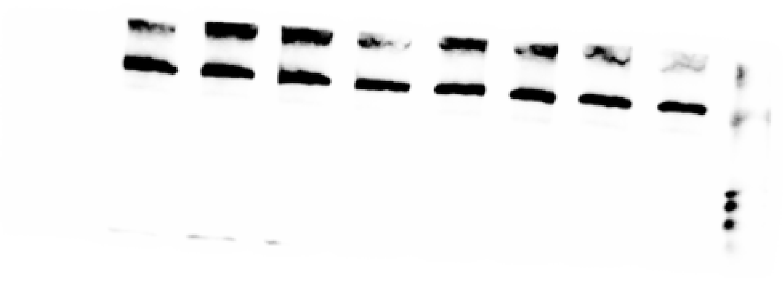

Raw data for Western Blots of DHODH(43KDa)

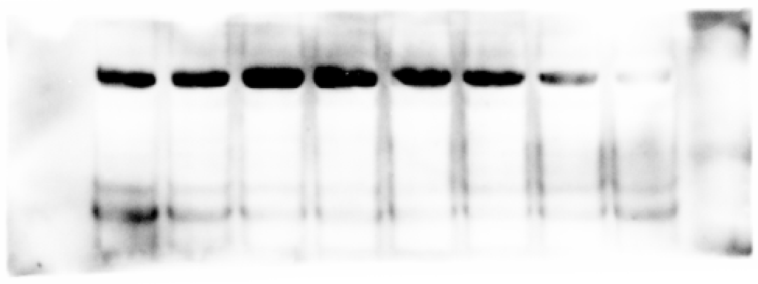

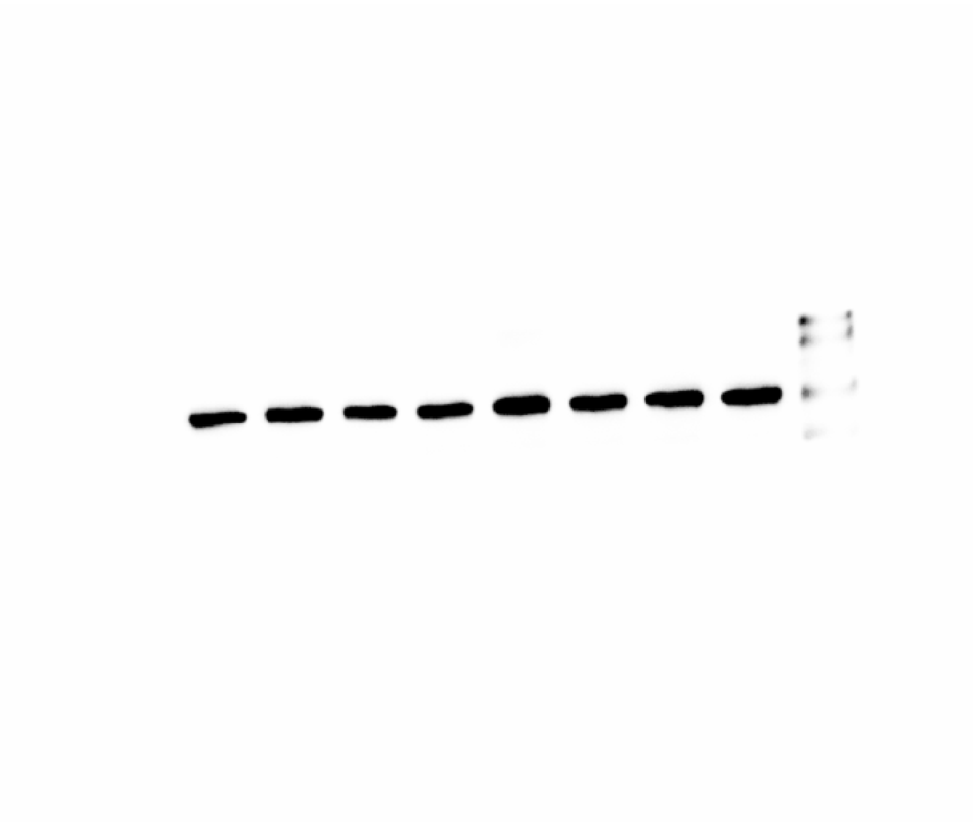

Raw data for Western Blots of PAICS(47KDa)

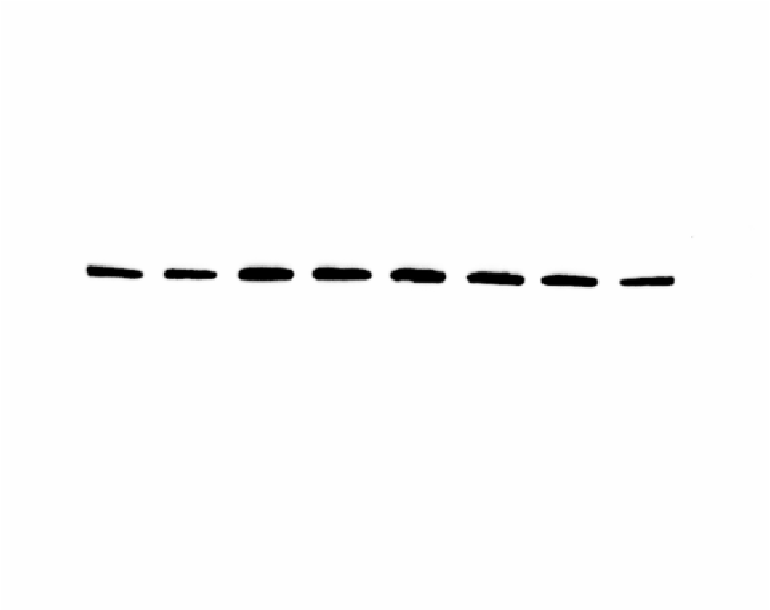

Raw data for Western Blots of β-actin(42KDa)

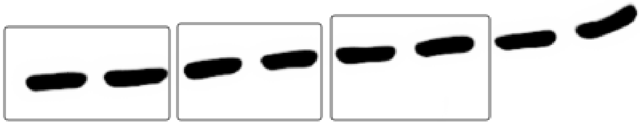

Supplement: bgae037_suppl_Supplementary_Materials [file bgae037_suppl_supplementary_materials.zip › suppl/Raw data for Western Blots.pdf]
