# Supplementary material for: Dehydroabietylamine exerts antitumor effects by affecting nucleotide metabolism in gastric cancer
Source: Carcinogenesis. 2024 Jun 13;45(10):759–72. doi: 10.1093/carcin/bgae037 (PMC11464700; doi:10.1093/carcin/bgae037)
Supplement: bgae037_suppl_Supplementary_Materials [file bgae037_suppl_supplementary_materials.zip › suppl/Supplementary Figure2.pdf]

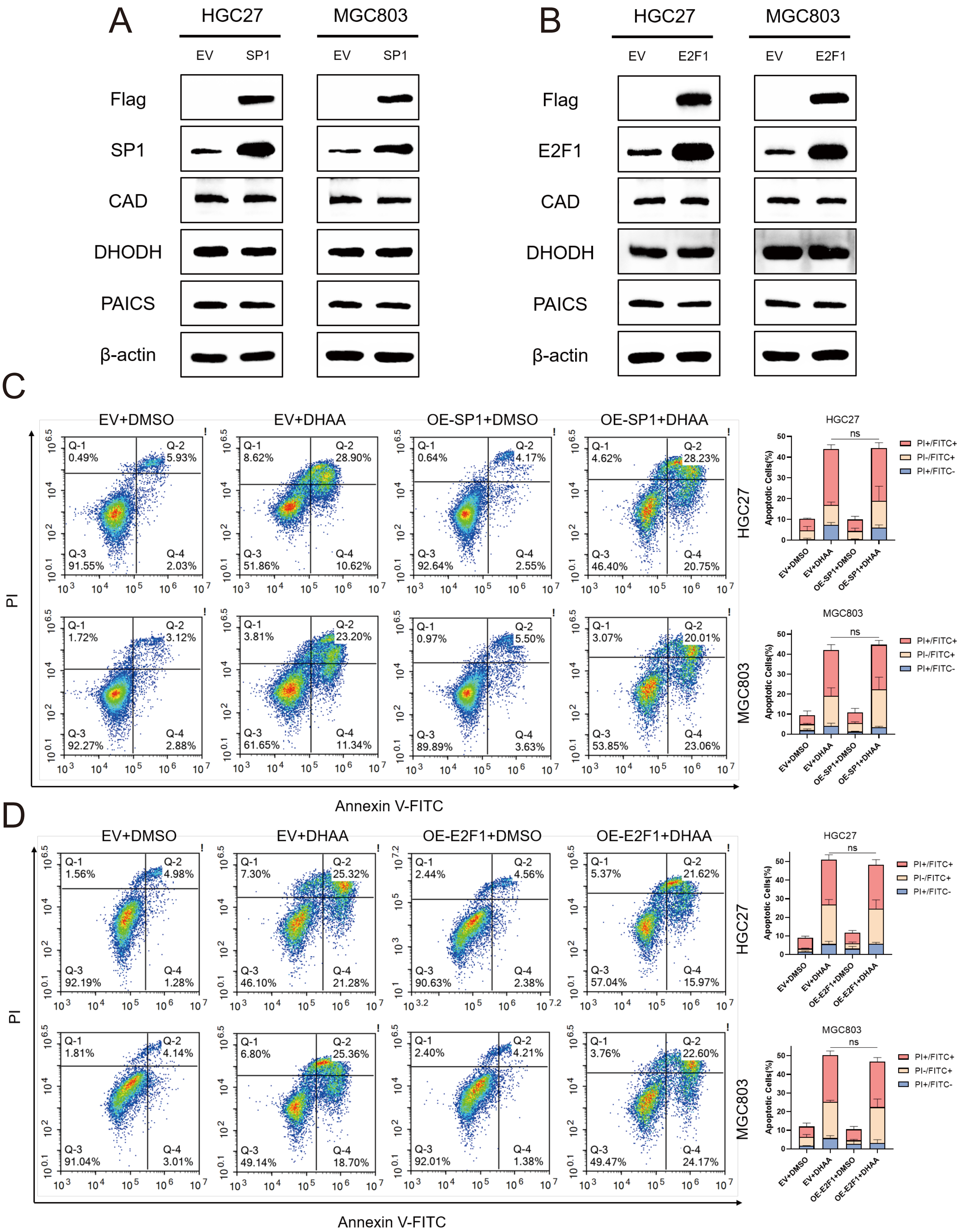

A
HGC27
MGC803
EV
SP1
Flag
SP1
CAD
DHODH
PAICS
β-actin
B
HGC27
MGC803
EV
E2F1
Flag
E2F1
CAD
DHODH
PAICS
β-actin
C
EV+DMSO
EV+DHAA
OE-SP1+DMSO
OE-SP1+DHAA
Q-1 0.49%
Q-2 5.93%
Q-3 91.55%
Q-4 2.03%
Q-1 8.62%
Q-2 28.90%
Q-3 51.86%
Q-4 10.62%
Q-1 0.64%
Q-2 4.17%
Q-3 92.64%
Q-4 2.55%
Q-1 4.62%
Q-2 28.23%
Q-3 46.40%
Q-4 20.75%
Q-1 1.72%
Q-2 3.12%
Q-3 92.27%
Q-4 2.88%
Q-1 3.81%
Q-2 23.20%
Q-3 61.65%
Q-4 11.34%
Q-1 0.97%
Q-2 5.50%
Q-3 89.89%
Q-4 3.63%
Q-1 3.07%
Q-2 20.01%
Q-3 53.85%
Q-4 23.06%
PI
Annexin V-FITC
HGC27
MGC803
Apoptotic Cells(%)
ns
PI+/FITC+
PI-/FITC+
PI+/FITC-
D
EV+DMSO
EV+DHAA
OE-E2F1+DMSO
OE-E2F1+DHAA
Q-1 1.56%
Q-2 4.98%
Q-3 92.19%
Q-4 1.28%
Q-1 7.30%
Q-2 25.32%
Q-3 46.10%
Q-4 21.28%
Q-1 2.44%
Q-2 4.56%
Q-3 90.63%
Q-4 2.38%
Q-1 5.37%
Q-2 21.62%
Q-3 57.04%
Q-4 15.97%
Q-1 1.81%
Q-2 4.14%
Q-3 91.04%
Q-4 3.01%
Q-1 6.80%
Q-2 25.36%
Q-3 49.14%
Q-4 18.70%
Q-1 2.40%
Q-2 4.21%
Q-3 92.01%
Q-4 1.38%
Q-1 3.76%
Q-2 22.60%
Q-3 49.47%
Q-4 24.17%
PI
Annexin V-FITC
HGC27
MGC803
Apoptotic Cells(%)
ns
PI+/FITC+
PI-/FITC+
PI+/FITC-

E

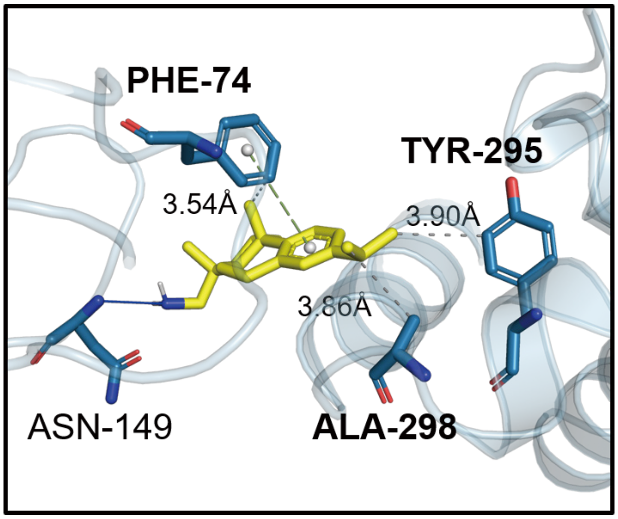

F

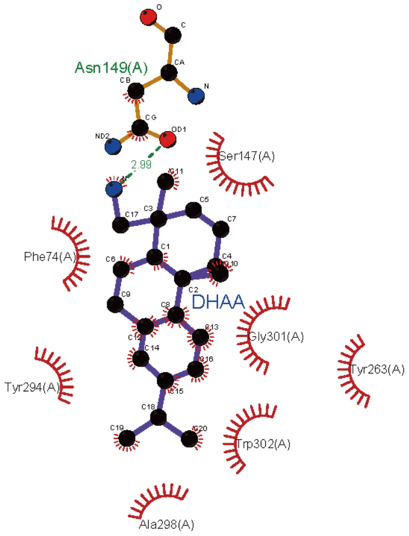

DHAA+FOXK2

G

Hydrophobic Interactions

Index	Residue	AA	Distance	Ligand Atom	Protein Atom
1	74A	PHE	3.54	10	510
2	295A	TYR	3.90	23	2173
3	298A	ALA	3.86	22	2199

Hydrogen Bonds

Index	Residue	AA	Distance H-A	Distance D-A	Donor Angle	Protein donor?	Side chain	Donor Atom	Acceptor Atom
1	149A	ASN	2.87	3.37	112.74	✔	✖	1071 [Nam]	18 [N3]

π-Stacking

Index	Residue	AA	Distance	Angle	Offset	Stacking Type	Ligand Atoms
1	74A	PHE	4.94	69.24	0.99	T	8, 12, 13, 14, 15, 16
