# Supplementary figures and images for: Dehydroabietylamine exerts antitumor effects by affecting nucleotide metabolism in gastric cancer
Source: Carcinogenesis. 2024 Jun 13;45(10):759–72. doi: 10.1093/carcin/bgae037 (PMC11464700; doi:10.1093/carcin/bgae037)

A

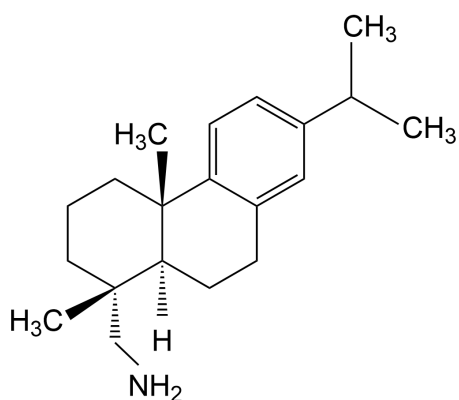

B

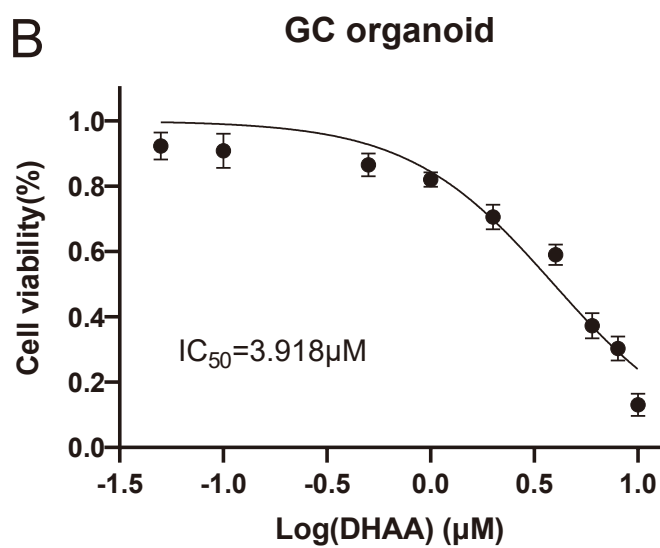

C

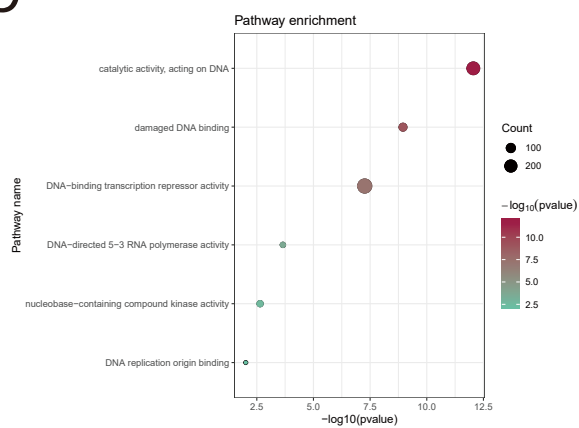

D

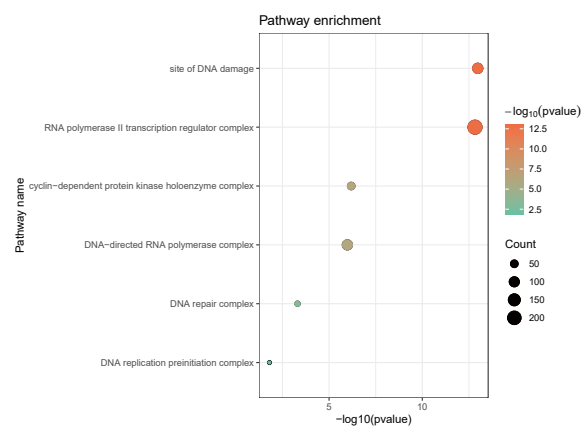

E

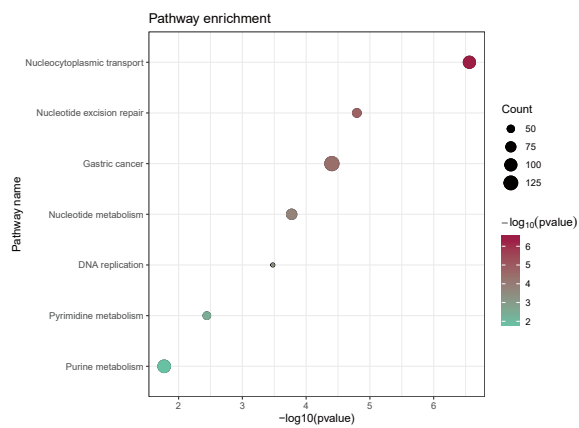

F

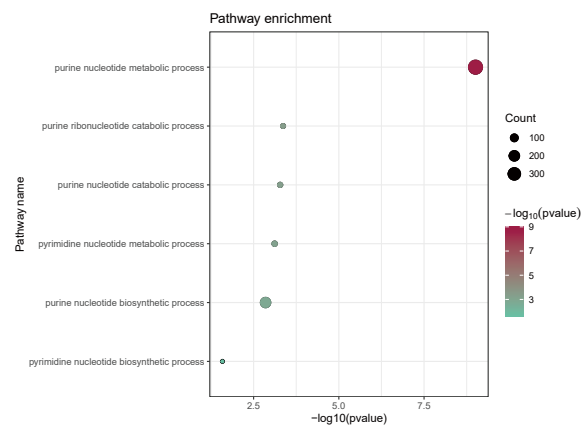

G

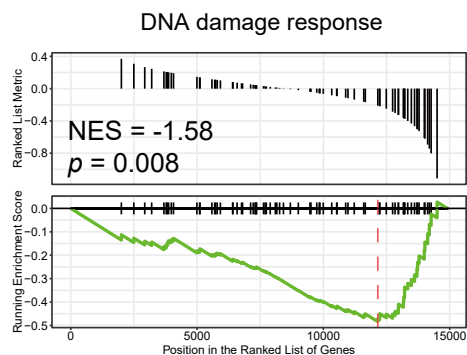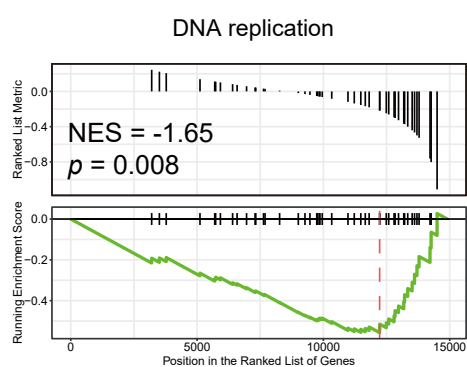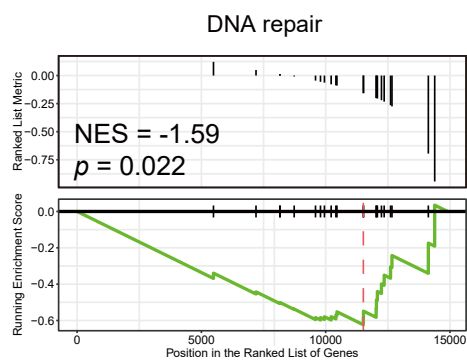

H

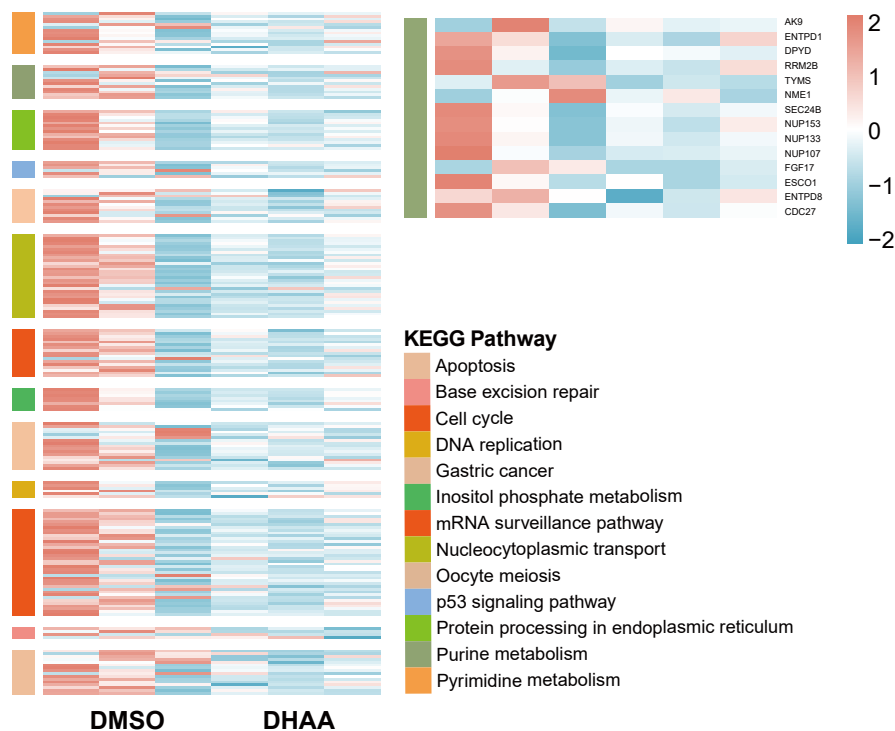

I

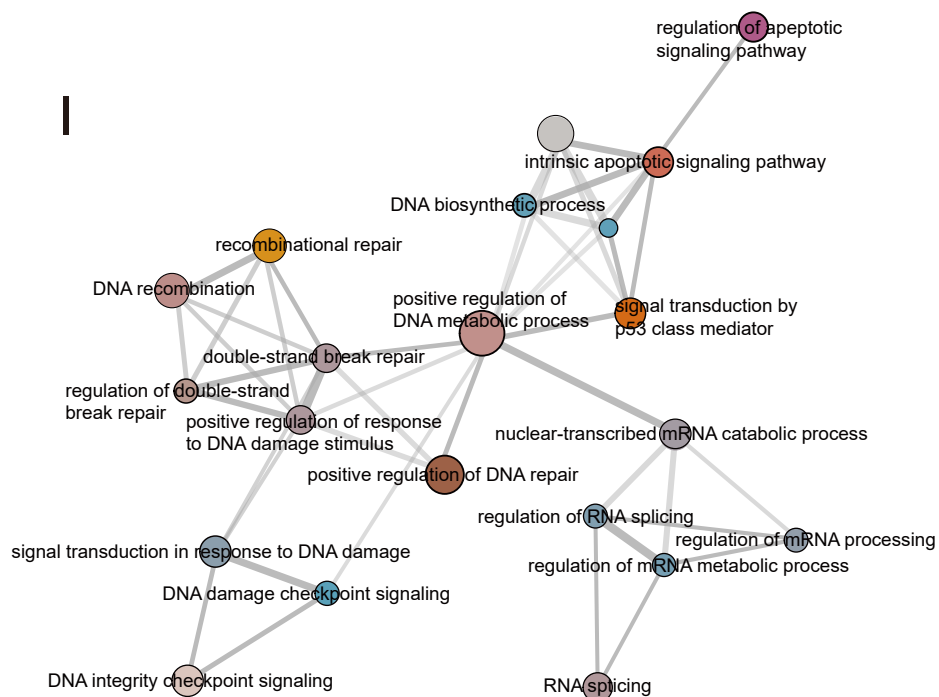

Supplement: bgae037_suppl_Supplementary_Materials [file bgae037_suppl_supplementary_materials.zip › suppl/Supplementary Figure1.pdf]
